# Associations of general and specific psychopathology factors by age 35 with dementia after age 50: a Swedish population-based sibling comparison study

**DOI:** 10.1038/s41380-026-03769-z

**Published:** 2026-07-24

**Authors:** Yangjun Liu, Sara Hägg, Isabell Brikell, Zheng Chang, Ralf Kuja-Halkola, Brian M. D’Onofrio, Henrik Larsson, Paul Lichtenstein, Erik Pettersson

**Affiliations:** 1 Department of Medical Epidemiology and Biostatistics, Karolinska Institutet, Stockholm, Sweden; 2 Department of Global Public Health and Primary Care, University of Bergen, Bergen, Norway; 3 Department of Biomedicine, Aarhus University, Aarhus, Denmark; 4 Department of Psychological and Brain Sciences, Indiana University, Bloomington, IN USA; 5 School of Medical Sciences, Örebro University, Örebro, Sweden

**Keywords:** Addiction, Bipolar disorder, Depression, Schizophrenia

## Abstract

Multiple psychiatric disorders are associated with later dementia, but it remains unclear whether these associations reflect a shared liability toward all psychiatric conditions (general psychopathology factor) or diagnosis-specific effects (specific psychopathology factors). In this Swedish register-based cohort study, we investigated these questions while adjusting for familial confounding shared by siblings (*N* = 2 543 621 individuals, 1 485 880 full-sibling pairs). Exposures were (i) six psychiatric diagnoses recorded by age 35 and (ii) a latent bifactor model fit to these diagnoses that identified one general and three specific (internalizing, externalizing, and psychotic) psychopathology factors. Outcomes were all-cause dementia and Alzheimer’s disease recorded after age 50. For observed psychiatric diagnoses, we estimated between-individual (HR) and within-sibling hazard ratios (HR_wn_) using Cox regression; for latent factors, we estimated between-individual (OR) and within-sibling odds ratios (OR_wn_) using exploratory structural equation modelling. All psychiatric diagnoses were significantly associated with increased risk of all-cause dementia (HR range 2.03–3.59; HR_wn_ range 1.72–2.94) and Alzheimer’s disease (HR range 1.89–3.47; HR_wn_ range 1.77–3.22). These associations were largely attributable to the general psychopathology factor, even after adjusting for familial confounding (OR_wn_ with 95% CI: 1.25 [1.17–1.32] for dementia; 1.24 [1.16–1.32] for Alzheimer’s disease). After accounting for the general factor, only the psychotic-specific factor remained associated (OR_wn_ with 95% CI: 1.20 [1.07–1.36] for dementia; 1.20 [1.06–1.37] for Alzheimer’s disease). These results suggest that liability toward general psychopathology and, independently, psychotic conditions, by early adulthood might be early markers of increased dementia risk and targets for timely identification and prevention.

## Introduction

Individuals with psychiatric disorders have a significantly higher risk of developing dementia later in life. For instance, in a nationwide cohort of 1.7 million New Zealand residents followed for up to three decades, any psychiatric diagnosis was associated with increased risk of both all-cause dementia and Alzheimer’s disease [1]. This increased risk seems to apply to most psychiatric conditions, including depression [2–5], anxiety [2, 6], post-traumatic stress disorder [7], obsessive-compulsive disorder [8], schizophrenia [2, 9], bipolar disorder [2, 10], substance misuse [11], and alcohol use disorder [2].

The mechanisms underlying the association between psychiatric conditions and later dementia remain unclear. One possibility is that these mechanisms are diagnosis-specific; for example, depression may be related to neuroinflammation, whereas alcohol use may be associated with altered brain development [12]. An alternative possibility, given that all psychiatric conditions tend to co-occur [13, 14], is that the risks are shared [12]. One way to test this is by applying so-called bifactor models [15], which partition the shared liability toward all psychiatric conditions (typically labeled general psychopathology factor, or p factor), from liabilities toward subsets of psychiatric conditions (e.g., specific internalizing, externalizing, and psychotic factors) [16, 17]. As the general psychopathology factor is associated with typical dementia risk factors such as general intelligence and brain health [17], it might also be prospectively related to dementia. Although the only study to date observed a null association between the general psychopathology factor and later dementia [18], it included a relatively small sample of older adults (*N* = 1072; aged 70–90 years), which potentially compromised power to detect even modest effects [18].

Additionally, prior research is limited in that the associations might be attributable to unmeasured shared familial factors, such as genetic vulnerability or early-life environments. Although a large twin study showed that the association between depression and later dementia remained significant after controlling for these factors [4], it remains unclear if associations with other psychiatric conditions persist after similar adjustment.

Further complicating matters, some prior studies had shorter follow-up periods, and evidence suggests that associations between psychiatric disorders and dementia weaken as the interval between diagnoses increases [3, 19]. Short follow-up may also increase the risk of misattributing early cognitive decline to psychiatric symptoms. Therefore, longer follow-up and extended time lags between psychiatric and dementia diagnoses may better clarify these associations.

To address these gaps, we conducted a nationwide, population-based cohort study using Swedish register data to examine whether the associations between psychiatric diagnoses by age 35 and dementia after age 50 were attributable to the general psychopathology factor, and we used a sibling comparison design to account for unmeasured familial confounding.

## Methods

### Participants

This Swedish nationwide population-based cohort study included two analytic samples. First, the full sample comprised 2 543 621 individuals born in Sweden between January 1, 1944, and December 31, 1968, who were alive, had not emigrated, and had no dementia diagnosis by age 50. These birth years ensured sufficient follow-up for psychiatric (exposure) and dementia (outcome) diagnoses, as the National Patient Register began in 1964 and achieved good nationwide inpatient coverage from 1973 [20]. Second, from this cohort, we identified a subsample of 1 485 880 unique full sibling pairs, drawn from 1 688 051 individuals across 678 561 family clusters (families with more than two children contributed multiple unique sibling pairs).

Data used in this study were obtained from the Swedish Medical Birth Register [21], Multi-Generation Register [22], National Patient Register [20], Cause of Death Register [23], and the Longitudinal Integrated Database for Health Insurance and Labour Market Studies (LISA) [24]. These registers were linked using the unique personal identification number assigned to all Swedish residents.

### Ethics approval and consent to participate

The study was approved by the Swedish Ethical Review Authority (Dnr 2020–06540). Informed consent was waived for the use of de-identified register data by law. All methods were performed in accordance with the relevant guidelines and regulations.

### Exposures

The exposures included (i) six psychiatric diagnoses, and (ii) one general psychopathology factor and three uncorrelated specific (internalizing, substance misuse, and psychotic) factors derived from a bifactor model based on these six diagnoses. The six psychiatric diagnoses included depression, anxiety spectrum disorder, drug use disorder, alcohol use disorder, schizophrenia, and bipolar disorder (relevant ICD codes are listed in Table S1 in Supplement). Diagnoses included in this study were selected based on prior literature linking them to dementia [2–11], and on the expectation that they would have sufficient prevalence. To ensure a sufficient (15-year) temporal separation between psychiatric and dementia diagnoses, we included psychiatric diagnoses recorded in the National Patient Register up to age 35. As the Outpatient Register begins in 2001, outpatient data by age 35 were available only for individuals born near the end of the inclusion period (1966–1968). Therefore, most psychiatric diagnoses were obtained from the Inpatient Register and thus primarily reflect more severe conditions. Both primary and secondary psychiatric diagnoses were used in this study.

### Outcomes

Outcomes were all-cause dementia and Alzheimer’s disease after age 50, identified as diagnoses in the National Patient Register or as underlying causes of death in the Cause of Death Register. Individuals were followed from age 50 until the first dementia diagnosis, death, emigration, or December 31, 2020, whichever occurred first. Age at the end of the follow-up ranged from 52–77 years.

All-cause dementia and Alzheimer’s disease were identified using ICD codes recorded as either primary or secondary diagnoses (Table S1 in Supplement). Validation studies indicate that dementia diagnoses in the Swedish National Patient and Cause of Death Registers have high positive predictive value but limited sensitivity [25], while subtype diagnoses such as Alzheimer’s disease have moderate validity [25–27]. Additionally, as dementia diagnoses are recorded in the National Patient Register about 5.5 years (95% CI: 5.5–5.8) after clinical onset [26], we identified outcomes after age 50 to ensure a minimum 15-year exposure-outcome lag, preserving an approximate 10-year gap even after accounting for diagnostic delay.

### Covariates

We included age at the end of follow-up, sex, and highest education as observed covariates.

### Statistical analysis

We examined associations between each psychiatric diagnosis and subsequent dementia using Cox regression in the full sample, with attained age as the time scale and adjustment for sex and highest education. We then used stratified Cox regression (i.e., a fixed effects model) in the full-sibling subsample, controlling for the observed covariates and all unmeasured confounders shared by siblings (e.g., ~50% genetics and 100% shared early-life environment).

To evaluate the extent to which the associations with dementia were accounted for by the shared liability toward all psychiatric disorders, we first performed an exploratory factor analysis of the six psychiatric diagnoses. Although the scree plot indicated a single factor model (eigenvalues: 3.48, 0.88, 0.68, 0.42, 0.34, and 0.20), we extracted two additional factors based on the theoretical notion that psychiatric phenomena are multidimensional, and that such specific factors might matter for later dementia (in the sensitivity analysis, we additionally explored lower-dimensional models). The resulting three-factor model fit the data well (Root Mean Square Error of Approximation [RMSEA] < 0.001; Comparative Fit Index [CFI] = 1; Tucker-Lewis Index [TLI] = 1; and Standardized Root Mean Square Residual [SRMR] = 0.003; Chi-square test of model fit: χ2 = 230 107.28, Degrees of Freedom = 15, *p* < 0.001). Next, we applied a Direct Schmid–Leiman bifactor rotation to these factors [28], identifying one general and three uncorrelated specific psychopathology factors (see Supplementary Methods in Supplement). The general factor captured the shared variance across all six diagnoses, while the specific factors captured variance unique to subsets of diagnoses over and above the general factor.

The dementia outcomes were then regressed onto the latent general and the three specific factors using Exploratory Structural Equation Modeling (ESEM) [29]. We first analyzed the full sample, adjusting for observed covariates, and then the full-sibling subsample that controlled for both the observed covariates and all unmeasured confounders shared by siblings (i.e., a fixed effects model) [30]. As Cox models are somewhat incompatible with latent variable modeling, we used a probit link and converted probit coefficients to odds ratios (ORs) for interpretability [OR=exp(probit coefficient × 1.6)] [31].

We performed the data extraction using SAS (version 9.4), the ESEM using Mplus (version 8.3; Muthén & Muthén) and the remaining analyses using R (version 4.5.1).

### Sensitivity analyses

We conducted ten sensitivity analyses to assess the robustness of our findings. First, to examine if the associations remained similar while making fewer assumptions than the latent fixed-effects model, we performed a sibling co-aggregation analysis, which examined whether psychiatric diagnoses and latent psychopathology factors in one sibling were associated with dementia outcomes in the other. Second, to address the effect of number of extracted factors, we reran the ESEM models using two alternative specifications: (i) extracting two latent factors and rotating them into one general and two uncorrelated specific factors using the Direct Schmid–Leiman transformation, and (ii) extracting a single general factor (without rotation). Third, because HRs could not be obtained from the ESEM analyses, which estimated ORs for associations between latent psychopathology factors and later dementia, we additionally estimated ORs using logistic regression for each psychiatric diagnosis, adjusting for the same covariates. This improved comparability across models and assessed whether conclusions were consistent when dementia was modeled as a binary rather than time-to-event outcome. Fourth, to further improve comparability across analytic approaches and to examine whether the results were robust to alternative measures of general psychopathology, we used three derived or observed proxies: (i) the factor score from the single general-factor model, (ii) a standardized first principal component score (mean = 0, SD = 1), and (iii) the total number of psychiatric diagnoses. We examined their associations with dementia outcomes using Cox regression, first in the full sample adjusting for observed covariates and then in the full-sibling subsample, additionally controlling for unmeasured confounders shared by siblings. Fifth, we evaluated consistency across samples by comparing between-individual associations in the full-sibling subsample with those in the full sample. Sixth, given known sex differences in the prevalence of psychiatric disorders and dementia, we tested sex stratification of associations in the full sample. Seventh, we explored if the associations differed between early-onset (before age 65) and late-onset (age 65 or older) dementia by conducting separate analyses for each outcome. Eighth, to examine the direction of potential selection bias introduced by conditioning on individuals who were alive, had not emigrated, and were dementia-free by age 50, we (i) compared the distribution of psychiatric diagnoses between those included in the final cohort and those excluded, and (ii) repeated the Cox regression analyses using the standardized first principal component score as the exposure under alternative conditioning criteria (alive, not emigrated, and dementia-free by ages 35, 40, and 45, respectively), and redefined psychiatric diagnoses as occurring by age 25 instead of age 35. Ninth, because educational attainment might lie on the pathway between psychopathology and subsequent dementia risk, we repeated the analyses without adjustment for highest educational level. Tenth, we examined whether additional psychiatric disorder groups not included in the main analyses were associated with subsequent dementia. These groups included physiological-disturbance disorders, personality disorders, developmental disorders, behavioral disorders, and unspecified mental disorders (ICD codes are presented in Table S2 in Supplement). However, because developmental disorders, behavioral disorders, and unspecified mental disorders had low prevalence, we examined associations for physiological-disturbance disorders, personality disorders, and a combined category including all five disorder groups. We applied the same statistical models as in the main analyses.

## Results

### Demographic characteristics

The full sample included 1 246 011 women (48.99%) and 1 297 610 men (51.01%). By age 35, the proportion of psychiatric diagnosis ranged from 0.16% (bipolar disorder) to 1.06% (alcohol use disorder). Median follow-up from age 50 was 13.96 years (interquartile range [IQR] 7.55–20.55). During follow-up, 23 410 individuals (0.92%) were diagnosed with dementia, including 18 628 (0.73%) with Alzheimer’s disease. Table S3 in Supplement presents onset age distributions for psychiatric and dementia diagnoses. The full-sibling subsample showed similar characteristics (Table 1 and Table S3 in Supplement). The number of sibling pairs discordant for both the exposure and the outcome ranged from 88 for bipolar disorder–Alzheimer’s disease to 1 947 for total number of psychiatric diagnoses–all-cause dementia (Table S4 in Supplement).

**Table 1:** Characteristics of participants and proportion of diagnoses in the two samples.

	Full sample (N = 2 543 621)	Full-sibling subsample (N = 1 688 051)
**Sex, n (%)**		
Female	1 246 011 (48.99)	825 749 (48.92)
Male	1 297 610 (51.01)	862 302 (51.08)
**Highest education level, n (%)**		
<=9 years	453 714 (17.84)	302 593 (17.93)
10–12 years	1 234 885 (48.55)	825 673 (48.91)
>=12 years	832 335 (32.72)	556 715 (32.98)
Missing	22 687 (0.89)	3 070 (0.18)
**Year of birth, n (%)**		
1944–1948	580 983 (22.84)	313 742 (18.59)
1949–1953	504 866 (19.85)	355 739 (21.07)
1954–1958	470 846 (18.51)	360 897 (21.38)
1959–1963	465 068 (18.28)	356 354 (21.11)
1964–1968	521 858 (20.52)	301 319 (17.85)
**Length of follow-up from age 50**		
Mean (SD)	14.08 (7.32)	13.94 (6.84)
Median (IQR)	13.96 (7.55, 20.55)	13.71 (8.12, 19.63)
**Age at the end of the follow-up**		
Mean (SD)	64.08 (7.32)	63.94 (6.84)
Median (IQR)	63.96 (57.55, 70.55)	63.71 (58.12, 69.63)
**Psychiatric diagnosis, n (%)**		
Depression	22 972 (0.90)	14 413 (0.85)
Anxiety spectrum disorder	25 311 (1.00)	15 457 (0.92)
Drug use disorder	14 632 (0.58)	8 623 (0.51)
Alcohol use disorder	26 944 (1.06)	16 374 (0.97)
Schizophrenia	8 151 (0.32)	5 205 (0.31)
Bipolar disorder	4 158 (0.16)	2 783 (0.16)
**All-cause dementia, n (%)**		
All	23 410 (0.92)	14 059 (0.83)
Early-onset	8 660 (0.34)	5 696 (0.34)
Late-onset	14 750 (0.58)	8 363 (0.5)
**Alzheimer’s disease, n (%)**		
All	18 628 (0.73)	11 126 (0.66)
Early-onset	6 766 (0.27)	4 436 (0.26)
Late-onset	11 862 (0.47)	6 690 (0.4)

*SD* standard deviation, *IQR* interquartile range.

### Association between psychiatric diagnoses and subsequent all-cause dementia and Alzheimer’s disease

**Fig. 1:**
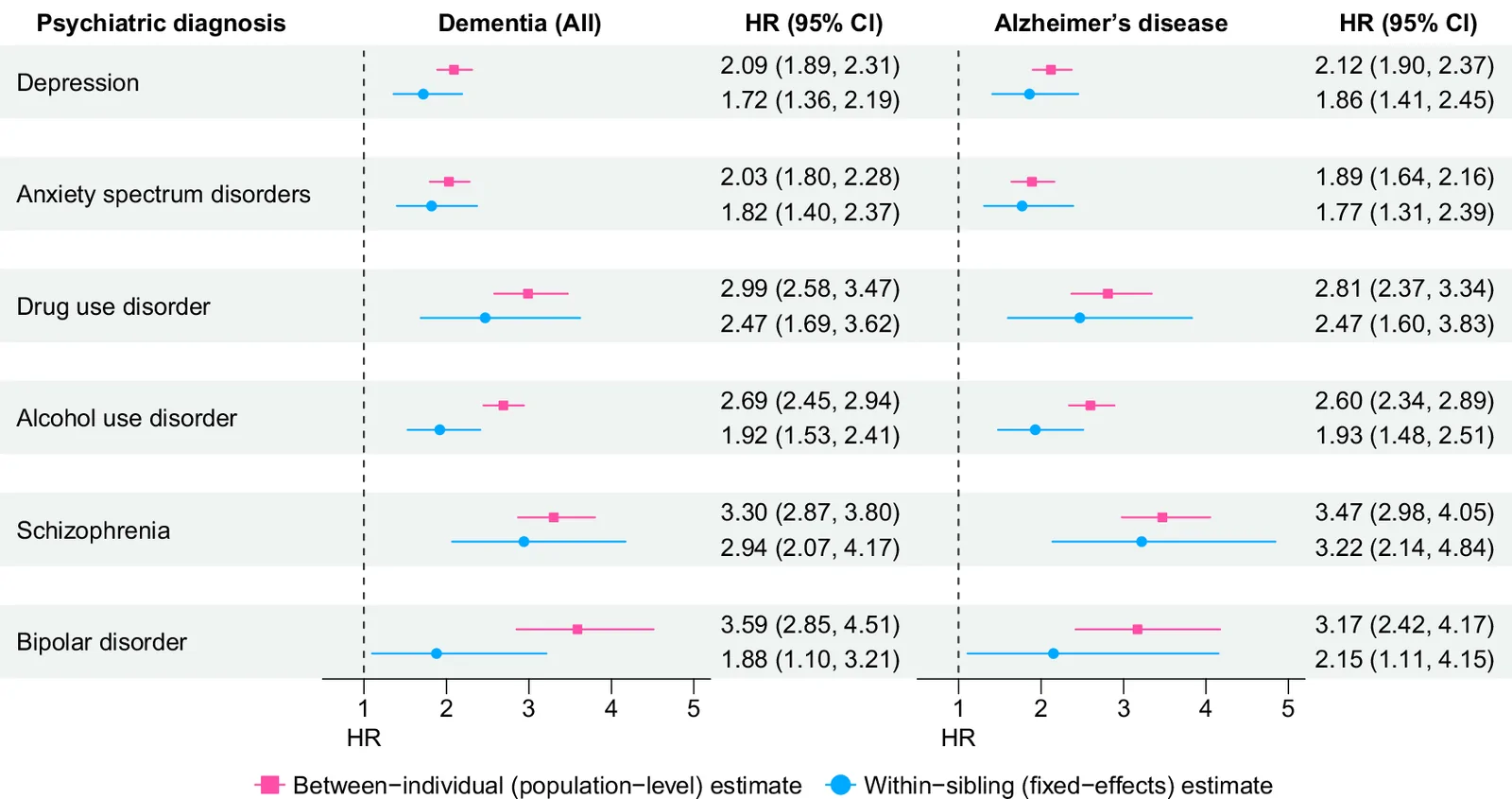
Associations between psychiatric diagnoses and subsequent all-cause dementia and Alzheimer’s disease. HR hazard ratio. Between-individual (population-level) estimates were derived from regressions applied to the full sample, adjusted for age, sex, and education level. Within-sibling (fixed-effects) estimates were derived from fixed-effects models applied to the full-sibling subsample, adjusting for both measured sibling-varying confounders (age, sex, and education level) and unmeasured sibling-shared confounders (i.e., approximately 50% genetics and 100% shared early-life environment).

As shown in Fig. 1, individuals with any of the six psychiatric diagnoses by age 35 had a significantly higher risk of all-cause dementia (HRs range: 2.03–3.59) and Alzheimer’s disease (HRs range: 1.89–3.47) after age 50. After further adjusting for unmeasured familial confounding, the associations attenuated mildly but remained statistically significant (HRs range: 1.72–2.94 for all-cause dementia, and 1.77–3.22 for Alzheimer’s disease).

### Association between latent psychopathology factors and subsequent all-cause dementia and Alzheimer’s disease

Table 2 displays factor loadings, with all psychiatric diagnoses loading positively on the general psychopathology factor (mean: 0.63; range: 0.56–0.68). The three specific factors captured internalizing (depression and anxiety spectrum disorder), substance misuse (drug use disorder and alcohol use disorder), and psychotic (schizophrenia and bipolar disorder) conditions.

**Table 2:** Exploratory factor analysis of the six psychiatric diagnoses.

	Rotation: Direct Schmid–Leiman			
	General psychopathology factor	Specific internalizing factor	Specific substance misuse factor	Specific psychotic factor
Depression	**0.67**	**0.54**	0.03	0.11
Anxiety spectrum disorder	**0.61**	**0.37**	0.15	0.08
Drug use disorder	**0.68**	0.00	**0.60**	0.08
Alcohol use disorder	**0.65**	0.18	**0.56**	−0.08
Schizophrenia	**0.56**	−0.05	0.10	**0.51**
Bipolar disorder	**0.59**	0.24	−0.10	**0.44**

Loadings greater than 0.30 are shown in bold for visual clarity.

**Fig. 2:**
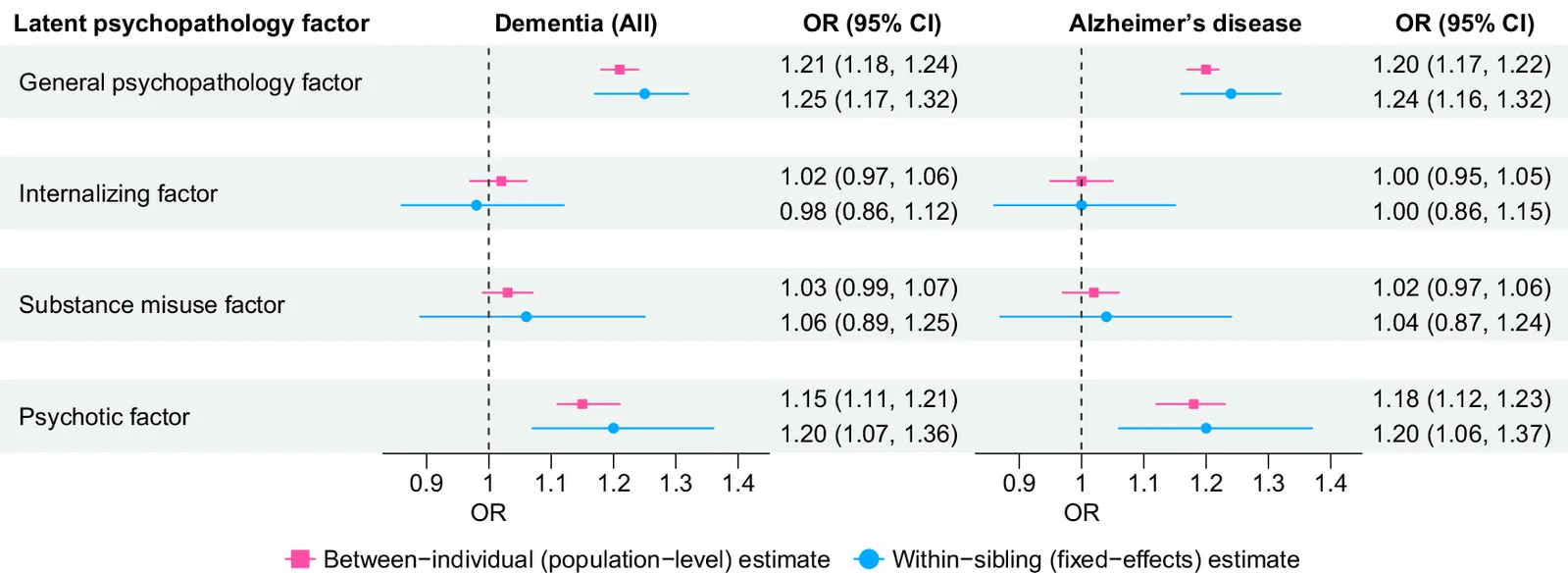
Associations between latent psychopathology factors and subsequent all-cause dementia and Alzheimer’s disease. OR odds ratio. Between-individual (population-level) estimates were derived from regressions applied to the full sample, adjusted for age, sex, and education level. Within-sibling (fixed-effects) estimates were derived from fixed-effects models applied to the full-sibling subsample, adjusting for both measured sibling-varying confounders (age, sex, and education level) and unmeasured sibling-shared confounders (i.e., approximately 50% genetics and 100% shared early-life environment).

As shown in Fig. 2, a one-standard-deviation increase in the latent general psychopathology factor was associated with increased risk of all-cause dementia (OR 1.21; 95% CI: 1.18–1.24) and Alzheimer’s disease (OR 1.20; 95% CI: 1.17–1.22). These associations remained after adjusting for unmeasured familial confounding (OR with 95% CI: 1.25 [1.17–1.32] for dementia; 1.24 [1.16–1.35] for Alzheimer’s disease).

After accounting for the general psychopathology factor, internalizing and substance misuse factors were not statistically significantly associated with all-cause dementia or Alzheimer’s disease (ORs range: 0.98–1.06). However, the psychotic factor remained associated with increased risk of dementias, even after controlling for unmeasured familial confounding (OR with 95% CI: 1.20 [1.07–1.36] for dementia; 1.20 [1.06–1.37] for Alzheimer’s disease).

### Sensitivity analyses results

The sensitivity analyses yielded similar results. First, consistent with the fixed effects results, the familial coaggregation analysis showed much weaker associations across siblings than those observed in the population-level analysis (Figures S1–S2 in Supplement), indicating that sibling-shared factors explained little of the association. Second, associations between the general psychopathology factor and dementia outcomes remained similar when fewer latent factors were extracted (Tables S5–S6; Figures S3–S4 in Supplement), indicating that the results were robust to the number of factors specified in the model. Third, odds ratios from logistic regression were broadly consistent in direction with the hazard ratios from the Cox models, supporting the overall pattern of findings, although the odds-ratio estimates were generally attenuated and some sibling fixed-effects associations were not statistically significant (Figure S5 in Supplement). Fourth, similar association patterns were observed using alternative derived or observed proxies for the general factor (Table S7; Figures S6–S8 in Supplement), implying that the associations were robust to alternative measures of general psychopathology factor and across analytic approaches. Fifth, between-individual associations in the full-sibling subsample closely mirrored those in the full sample (Figure S9 in Supplement), supporting the representativeness of the full-sibling subsample. Sixth, between-individual associations were comparable between males and females (Figures S10–S11 in Supplement), with no evidence of an interaction (all *p*-values > 0.05). Seventh, similar patterns were observed for early-onset and late-onset dementias, though with slightly stronger associations for early-onset outcomes (Figures S12–S13 in Supplement). Eighth, individuals and sibling pairs excluded from the final cohort were more likely to have psychiatric diagnoses or higher scores on the first principal component (Tables S8–S9 in the Supplement), indicating that those included in the final cohort were relatively healthier. This suggests that the true associations may be underestimated due to conditioning on being alive, having not emigrated, and remaining dementia-free by age 50. Consistent with this interpretation, associations were stronger when conditioning on earlier ages (35, 40, and 45 years), where such selection bias is less pronounced, and remained robust when defining psychiatric diagnoses by age 25 (Figure S14 in the Supplement). Ninth, analyses without adjustment for highest educational level yielded results similar to those of the main analyses (Figures S15–S16 in Supplement). This suggests that adjustment for educational attainment did not materially influence the observed associations. Tenth, physiological-disturbance disorders, personality disorders, and the combined category of additional psychiatric disorders showed associations with subsequent dementia that were broadly similar to those observed in the main analyses (Figure S17 in Supplement).

## Discussion

In this Swedish register-based study, whereas psychiatric diagnoses by age 35 predicted increased risk of all-cause dementia and Alzheimer’s disease after age 50 within sibling pairs, these associations appeared to be largely attributable to a shared liability toward all psychiatric diagnoses. Additionally, the psychotic-specific factor also predicted dementia over and above this general liability.

Our findings replicate prior evidence that psychiatric disorders are associated with increased dementia risk [1–11] and extend it by showing that this risk appeared to be largely explained by a shared liability toward all psychiatric conditions. Although the only prior study to date found no significant association between general psychopathology and dementia [18], this discrepancy may be due to differences in sample size (2 543 621 vs. 1 072), study population (individuals who were dementia-free at age 50 vs. 70) or timing of psychopathology assessment (age ≤ 35 vs. 70–90 years). One speculation is that psychiatric disorders in childhood, adolescence, and early adulthood may have more persistent and pronounced effects on brain and cognitive function, as this period is characterized by heightened neural plasticity and vulnerability [32, 33]. The discrepancy may also reflect that the prior study used self- and informant-report measures that potentially captured milder symptoms, whereas our register-based inpatient diagnoses primarily captured more severe conditions. In light of this, extrapolation of the current results to milder cases warrants caution.

We additionally observed that the associations between general psychopathology factor and later dementia remained statistically significant and of similar magnitude after adjustment for unmeasured familial confounding shared by siblings. Prior studies have shown that general psychopathology is associated with reduced cortical and gray matter volumes, lower neocortical thickness, and impaired myelin maturation in white matter tracts during youth and midlife [34–36], which are established predictors of cognitive impairment and dementia [37, 38]. Although the mechanisms remain unclear, one speculation is that elevated general psychopathology may reflect neurodevelopmental or neurobiological profiles associated with lower cognitive reserve or increased vulnerability to age-related neurodegeneration, contributing to dementia risk independently of shared familial or genetic liability. Alternatively, the association may reflect individual-specific factors not shared between siblings, including non-shared inherited genetic variation, de novo mutations, and modifiable environmental or lifestyle factors such as physical inactivity, smoking, and excessive alcohol use [39].

In addition, we observed an association between psychotic psychopathology factor and later dementia, above and beyond the effect of the general psychopathology factor. Although prior research indicates that psychotic psychopathology and dementia share genetic underpinnings [40, 41], in our study, the association remained statistically significant in within-sibling analyses, suggesting that shared familial factors, including genetic influences shared by siblings, did not fully explain the association. The underlying mechanism is unclear, but severe mental illnesses might be associated with earlier cognitive decline and neuroimaging patterns resembling older age (e.g., cortical thinning, reduced hippocampal and prefrontal volumes, and white-matter abnormalities) via non-genetic pathways [42–44]. Additionally, long-term antipsychotic exposure might also play a role, although the evidence is mixed [45].

Although these findings point to possible non-genetic pathways linking general and psychotic psychopathology factors with later dementia, it is important to note that the magnitudes of the observed associations were modest. In the same sibling fixed-effects models, the ORs for the general and psychotic-specific psychopathology factors were approximately 1.2 per 1-SD increase, compared with approximately 2.0 per 1-SD increase for age, one of the strongest predictors of dementia. However, the effect sizes were comparable to that reported for low-density lipoprotein, a modifiable predictor of dementia [39], with an OR of approximately 1.3 per 1-SD increase [46].

These findings have implications for risk stratification and prevention. For risk stratification, measuring a broader liability toward most psychiatric conditions, such as with the Hierarchical Taxonomy of Psychopathology (HiTOP) model [47], might improve early identification of individuals at increased risk of later-life dementia, beyond disorder-specific diagnoses. In terms of prevention, the significant within-sibling associations suggest potentially modifiable pathways independent of shared familial factors and support a shift toward transdiagnostic approaches targeting shared mechanisms across psychiatric conditions [48, 49]. For instance, as both general psychopathology and dementia are associated with indicators of poor physical health [39, 50], one speculation is that promoting healthy behaviors among those with a high liability toward several psychiatric conditions could boost overall brain health. Furthermore, future studies could explore whether the association between general psychopathology factor and later dementia is partly mediated through poor cardiometabolic or cardiovascular health, which might help identify potential intervention targets.

These results should be interpreted in light of several limitations. First, although we adjusted for both observed covariates (e.g., sex) and unmeasured shared familial confounding, associations may still reflect residual genetic confounding (as full siblings share on average only half of their segregating genetic variants) and other unmeasured individual-specific confounders not shared between siblings [51]. Second, psychiatric diagnoses were primarily obtained from inpatient specialist care, likely leading to under-ascertainment of milder cases, as reflected by the low prevalence of recorded psychiatric diagnoses. Therefore, the findings may be less generalizable to individuals with milder, untreated, or primary-care-managed psychiatric disorders. This low prevalence also suggests that some siblings with these conditions might have been misclassified as unexposed, which could bias estimated associations toward the null [52]. Furthermore, the low prevalence precluded analyses of more fine-grained psychiatric conditions (e.g., separating anxiety from fear-based conditions) and of some uncommon psychiatric conditions because of insufficient statistical power. Third, dementia diagnoses are often recorded with delay in the registers [26]. However, requiring a ≥ 15-year interval between psychiatric and dementia diagnoses likely reduced reverse causation and misclassification of prodromal dementia as psychiatric disorders. Moreover, the association between the general psychopathology factor and late-onset dementia remained significant even with a ≥ 30-year interval between exposure and outcome, further supporting the robustness of the findings. Fourth, medication data were not included because the Prescribed Drug Register began in 2005 and lacks sufficient longitudinal coverage; thus, although most patients admitted for inpatient care likely received psychotropic medications, we could not account for variation in treatment duration or intensity, which may influence dementia risk directly or via symptom course, potentially affecting the magnitude of the associations. Fifth, conditioning on survival, non-emigration, and being dementia-free at age 50 may introduce selection bias, potentially limiting generalizability and attenuating the observed associations, as suggested by our sensitivity analyses. Finally, as this study was based on Swedish registers, generalizability to other populations and healthcare systems may be restricted.

In conclusion, this study found that associations between psychiatric disorders by age 35 and all-cause dementia and Alzheimer’s disease after age 50 were largely attributable to a shared liability toward all psychiatric conditions (the general psychopathology factor), alongside an independent psychotic-specific factor. As these associations persisted after adjustment for familial confounding shared by siblings, liability toward general psychopathology and, independently, psychotic conditions, by early adulthood might be markers of later dementia risk and potential targets for early identification and prevention.

## Supplementary information

Supplementary Material

## Data Availability

The Swedish data used in this study cannot be made publicly available due to privacy regulations. However, researchers can apply for access to these confidential data from the register holder, the Swedish National Board of Health and Welfare.

## Code availability

Code used for the analyses is available from the corresponding author upon reasonable request.
