# Supplementary material for: Associations of general and specific psychopathology factors by age 35 with dementia after age 50: a Swedish population-based sibling comparison study
Source: Mol Psychiatry. 2026 Jul 24;31(12):7170–7. doi: 10.1038/s41380-026-03769-z (PMC13736510; doi:10.1038/s41380-026-03769-z)
Supplement: Supplementary file 1 [file 41380_2026_3769_MOESM1_ESM.pdf]

Supplementary Online Content

Article title

Associations of General and Specific Psychopathology Factors by Age 35 with Dementia after Age 50: A Swedish Population-Based Sibling Comparison Study

Authors

Yangjun Liu, MD, PhD[1]; Sara Hägg, PhD[1]; Isabell Brikell, PhD[1,2,3]; Zheng Chang, PhD[1]; Ralf Kuja-Halkola, PhD[1]; Brian M D'Onofrio, PhD[1,4]; Henrik Larsson, PhD[1,5]; Paul Lichtenstein, PhD[1]; Erik Pettersson, PhD[1]

Affiliations

[1]Department of Medical Epidemiology and Biostatistics, Karolinska Institutet, Stockholm, Sweden
[2]Department of Global Public Health and Primary Care, University of Bergen, Bergen, Norway
[3]Department of Biomedicine, Aarhus University, Aarhus, Denmark
[4]Department of Psychological and Brain Sciences, Indiana University, Bloomington, Indiana, USA
[5]School of Medical Sciences, Örebro University, Örebro, Sweden.

Table of Contents

Supplementary Methods: Direct Schmid–Leiman Transformation 3–4

Table S1. International Classification of Diseases (ICD) codes for diagnoses included in the main analysis 5

Table S2. ICD codes for additional psychiatric disorder groups included in the sensitivity analysis.................... 6

Table S3. Age at diagnosis for psychiatric disorders and dementia outcomes 7

Table S4. Number of sibling pairs discordant for both the exposure and the outcome 8

Table S5. Factor loadings of the six psychiatric diagnoses on one general and two specific latent factors 9

Table S6. Factor loadings of the six psychiatric diagnoses on one latent general factor 10

Table S7. Distribution of the total number of psychiatric diagnoses in the full sample and the full-sibling subsample 11

Table S8. Comparison between individuals included and excluded from the final full sample 12

Table S9. Comparison between sibling pairs included and excluded from the final full-sibling subsample........ 13

Figure S1. Between-individual and between-sibling associations of psychiatric diagnoses with subsequent all-cause dementia and Alzheimer's disease 14

Figure S2. Between-individual and between-sibling associations of latent psychopathology factors with subsequent all-cause dementia and Alzheimer's disease 15

Figure S3. Associations of three (one general and two specific) latent psychopathology factors with subsequent all-cause dementia and Alzheimer's disease 16

Figure S4. Associations of one latent general psychopathology factor with subsequent all-cause dementia and Alzheimer's disease 17

Figure S5. Odds ratios for the associations of psychiatric diagnoses with subsequent all-cause dementia and Alzheimer's disease 18

Figure S6. Associations of the factor score from the single general-factor model with subsequent all-cause dementia and Alzheimer's disease 19

Figure S7. Associations of the standardized score of the first principal component with subsequent all-cause dementia and Alzheimer's disease 20

Figure S8. Associations of the total number of psychiatric diagnoses with subsequent all-cause dementia and Alzheimer's disease 21

Figure S9. Between-individual associations of psychiatric diagnoses with subsequent all-cause dementia and Alzheimer's disease in the full sample and the full-sibling subsample 22

Figure S10. Between-individual associations of psychiatric diagnoses with subsequent all-cause dementia and Alzheimer's disease in males and females.. 23

Figure S11. Between-individual associations of latent psychopathology factors with subsequent all-cause dementia and Alzheimer's disease in males and females 24

Figure S12. Associations of psychiatric diagnoses with subsequent early-onset and late-onset all-cause dementia and Alzheimer's disease 25

Figure S13. Associations of latent psychopathology factors with subsequent early-onset and late-onset all-cause dementia and Alzheimer's disease 26

Figure S14. Associations of the standardized score of the first principal component by age 35 or 25 years with subsequent all-cause dementia and Alzheimer's disease after age 35, 40, 45, or 50 years.................................. 27

Figure S15. Associations between psychiatric diagnoses and subsequent all-cause dementia and Alzheimer's disease (without adjustment for educational level)... 28

Figure S16. Associations between latent psychopathology factors and subsequent all-cause dementia and Alzheimer's disease (without adjustment for educational level) 29

Figure S17. Associations between additional psychiatric disorder groups and subsequent all-cause dementia and Alzheimer's disease 30

Supplementary Methods: Direct Schmid–Leiman Transformation

We applied a Direct Schmid–Leiman (SL) bifactor transformation to separate the shared variance from domain-specific variance.[1, 2] The SL transformation reallocates the variance contained in the extracted factors into two distinct components:

1. variance shared across all factors, and
2. variance unique to each factor.

The shared variance is extracted as a single general factor (e.g., general psychopathology factor), and the remaining domain-specific variance from each original factor becomes corresponding specific factors (e.g., specific internalizing, substance misuse, and psychotic factors).

In our study, although we extracted three factors at the beginning, this decomposition increased the number of factors from three to four because the SL transformation separates *shared* and *unique* variance into distinct orthogonal factors. However, no new variance is introduced; the original variance is simply reorganized so that the general and specific components can be examined independently. For a detailed description of this process, including the analysis code, please refer to the supplementary methods section of Chen and colleagues.[3]

Illustration of the factor structure after the Direct Schmid–Leiman transformation

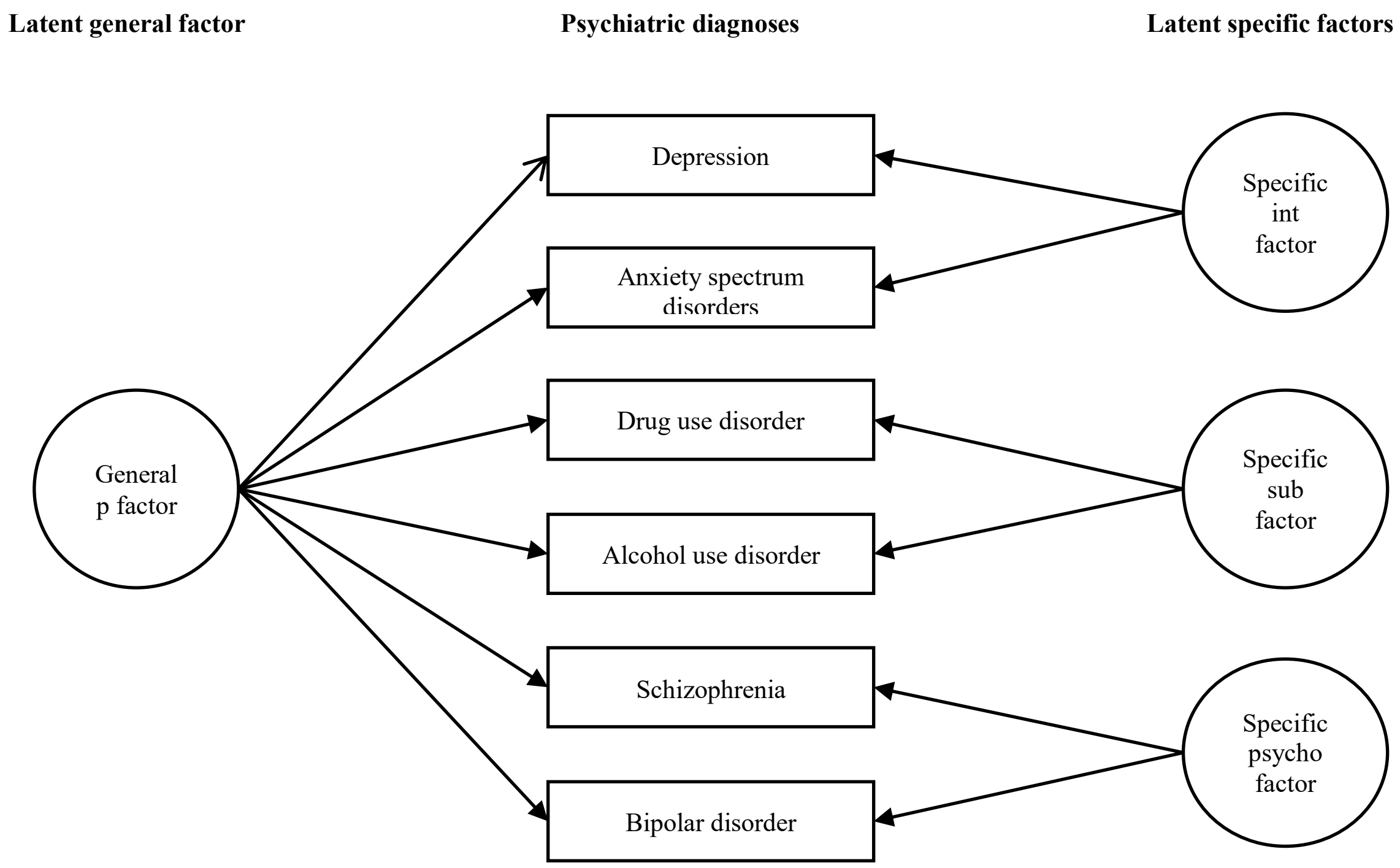

Note. The arrow lines from latent factors to indicators with loadings ≤ 0.3 were omitted in the figure. General p factor represents the general psychopathology factor. Specific int factor represents specific internalizing factor. Specific sub factor represents specific substance misuse factor. Specific psycho factor represents specific psychotic factor.

Supplementary References

1. Waller NG. Direct Schmid–Leiman Transformations and Rank-Deficient Loadings Matrices. Psychometrika 2018;83(4):858-870. DOI: 10.1007/s11336-017-9599-0.

2. Giordano C, Waller NG. Recovering bifactor models: A comparison of seven methods. Psychological Methods 2020;25(2):143-156. DOI: 10.1037/met0000227.

3. Chen C, Chang Z, Kuja-Halkola R, et al. Associations Between General and Specific Mental Health Conditions in Young Adulthood and Cardiometabolic Complications in Middle Adulthood: A 40-Year Longitudinal Familial Coaggregation Study of 672,823 Swedish Individuals. Am J Psychiatry. 2024 Jul 1;181(7):651-657. doi: 10.1176/appi.ajp.20220951.

Table S1. International Classification of Diseases (ICD) codes for diagnoses included in the main analysis

Diagnosis	ICD-10	ICD-9	ICD-8
Depression	F32-F39	311; 300E	300.4
Anxiety spectrum disorder	F40-45, F48	300 (excl. 300E)	300 (excl. 300.4)
Drug use disorder	F11-19 (excl. F11.5, F12.5, F13.5, F14.5, F15.5, F16.5, F17, F18.5, F19.5)	292, 304, 305X	304
Alcohol use disorder	F10 (excl. F10.5)	291, 303, 305A	291, 303
Schizophrenia	F20, F25	295A-295E, 295G, 295W, 295X, 295H	295.0-295.4, 295.6, 295.8, 295.9, 295.7
Bipolar disorder	F30, F31	296A-296E, 296W, 296X	296.0-296.3, 296.8, 296.9
All-cause dementia	F00, F01, F02, F03, F051, G30, G311, G318	290A, 290B, 290E, 290W, 290X, 294B, 331A, 331B, 331C, 331X	290, 2930, 2931
Alzheimer's disease	F00, F03, G30	290A, 290B, 290X, 331A	290

Note. ICD-8 and ICD-9 diagnoses were coded using the Swedish versions which are not fully consistent with the international versions.

Table S2. ICD codes for additional psychiatric disorder groups included in the sensitivity analysis

Mental disorders	Abbreviated names	ICD-10	ICD-9	ICD-8
Behavioral disorders associated with physiological disorders and physical factors	Physiological-disturbance disorders	F50 – F59	302, 305(excl. 305A, 305X), 306, 307 (excl.307X), 783A	302, 305, 306, 307, 784.0
Personality disorders and behavioral disorders in adults	Personality disorders	F60 – F69	301	301
Disturbances of mental development	Developmental disorders	F80 – F89	299, 315	/
Behavioral disorders and emotional disorders with onset usually during childhood and adolescence	Behavioral disorders	F90 – F98	312, 313, 314	308
Unspecified mental disorder	Unspecified mental disorders	F99	300X, 307X	/

Note. ICD-8 and ICD-9 diagnoses were coded using the Swedish versions which are not fully consistent with the international versions. For the physiological-disturbance disorders, ICD-9 codes 305A and 305X were excluded to avoid overlap, as they were already included in the main analysis.

Table S3. Age at diagnosis for psychiatric disorders and dementia outcomes

	Full sample (N = 2543621)		Full-sibling subsample (N = 1688051)	
	**Mean (SD)**	**Median (IQR)**	**Mean (SD)**	**Median (IQR)**
**Age of psychiatric disorder**				
Depression	27.25 (5.22)	27.94 (23.56, 31.61)	27.06 (5.19)	27.62 (23.26, 31.41)
Anxiety spectrum disorder	27.14 (6.05)	28.35 (23.39, 32.14)	27.15 (5.92)	28.29 (23.4, 32.06)
Drug use disorder	26.74 (5.03)	26.93 (22.81, 31.02)	26.72 (5.05)	26.89 (22.76, 31.03)
Alcohol use disorder	28.15 (4.52)	28.74 (24.89, 31.92)	28.01 (4.54)	28.53 (24.69, 31.84)
Schizophrenia	26.85 (4.77)	27.02 (23.17, 30.80)	26.63 (4.78)	26.6 (22.92, 30.62)
Bipolar disorder	28.96 (4.32)	29.72 (26.09, 32.46)	28.92 (4.4)	29.73 (26.04, 32.47)
**Age of dementia outcomes**				
All-cause dementia	66.59 (6.38)	67.79 (62.10, 71.74)	66.03 (6.38)	67.02 (61.44, 71.16)
Early-onset	59.45 (3.91)	60.11 (56.67, 62.77)	59.43 (3.9)	60.08 (56.64, 62.74)
Late-onset	70.61 (3.00)	70.63 (68.16, 73.02)	70.36 (2.99)	70.32 (67.9, 72.73)
Alzheimer's disease	66.74 (6.36)	67.94 (62.26, 71.89)	66.18 (6.36)	67.17 (61.57, 71.34)
Early-onset	59.50 (3.86)	60.19 (56.75, 62.76)	59.48 (3.85)	60.16 (56.72, 62.73)
Late-onset	70.66 (3.01)	70.73 (68.20, 73.10)	70.42 (3)	70.45 (67.92, 72.82)

Note. SD: standard deviation. IQR: interquartile range.

Table S4. Number of sibling pairs discordant for both the exposure and the outcome

Exposure	Outcome	
	**All-cause dementia, N**	**Alzheimer's disease, N**
Depression	601	484
Anxiety spectrum disorder	521	408
Drug use disorder	274	205
Alcohol use disorder	762	568
Schizophrenia	325	264
Bipolar disorder	123	88
Total number of psychiatric diagnoses	1947	1489

Table S5. Factor loadings of the six psychiatric diagnoses on one general and two specific latent factors

Psychiatric diagnosis	Rotation: Direct Schmid–Leiman		
	General psychopathology factor	Specific internalizing-psychotic factor	Specific substance misuse factor
Depression	**0.61**	**0.54**	0.07
Anxiety spectrum disorder	**0.59**	**0.44**	0.14
Drug use disorder	**0.67**	0.12	**0.55**
Alcohol use disorder	**0.68**	0.14	**0.55**
Schizophrenia	**0.45**	**0.34**	0.11
Bipolar disorder	**0.48**	**0.55**	-0.07

Note. Loadings greater than 0.30 are shown in bold. Two factors were extracted using exploratory factor analysis, and these two factors were rotated to one general and two specific factors using Direct Schmid–Leiman transformation. Model fit: Root Mean Square Error of Approximation (RMSEA) = 0.010; Comparative Fit Index (CFI) = 0.996; Tucker-Lewis Index (TLI) = 0.984; and Standardized Root Mean Square Residual (SRMR) = 0.037; Chi-square test of model fit: $\chi 2$ = 217582.309, Degrees of Freedom = 15, $p < 0.001$.

Table S6. Factor loadings of the six psychiatric diagnoses on one latent general factor

Psychiatric diagnosis	General psychopathology factor
Depression	**0.73**
Anxiety spectrum disorder	**0.72**
Drug use disorder	**0.85**
Alcohol use disorder	**0.83**
Schizophrenia	**0.55**
Bipolar disorder	**0.64**

Note. Loadings greater than 0.30 are shown in bold. One general psychopathology factor was extracted using exploratory factor analysis and no rotation was applied. Model fit: Root Mean Square Error of Approximation (RMSEA) = 0.016; Comparative Fit Index (CFI) = 0.974; Tucker-Lewis Index (TLI) = 0.956; and Standardized Root Mean Square Residual (SRMR) = 0.078; Chi-square test of model fit: $\chi 2$ = 217582.309, Degrees of Freedom = 15, $p < 0.001$.

Table S7. Distribution of the total number of psychiatric diagnoses in the full sample and the full-sibling subsample

Total number of psychiatric diagnoses, n (%)	Full sample (N = 2543621)	Full-sibling subsample (N = 1688051)
0	2465725 (96.94)	1639935 (97.15)
1	58375 (2.29)	36228 (2.15)
2	15546 (0.61)	9502 (0.56)
3	3276 (0.13)	1972 (0.12)
4	629 (0.02)	367 (0.02)
5	63 (0.002)	43 (0.003)
6	7 (0.0003)	<5 (<0.0003)

Note. Six psychiatric diagnoses were counted, which were depression, anxiety spectrum disorder, drug use disorder, alcohol use disorder, schizophrenia, and bipolar disorder. Numbers <5 were presented as "<5" due to data privacy policy.

Table S8. Comparison between individuals included and excluded from the final full sample

Number of individuals, n (%)	Individuals excluded (N=297387)	Individuals included in the final full sample (N=2543621)	*p* value
**Depression**			
No	292443 (98.34)	2520649 (99.10)	< 0.001
Yes	4944 (1.66)	22972 (0.90)	
**Anxiety spectrum disorder**			
No	292657 (98.41)	2518310 (99.00)	< 0.001
Yes	4730 (1.59)	25311 (1.00)	
**Drug use disorder**			
No	290400 (97.65)	2528989 (99.42)	< 0.001
Yes	6987 (2.35)	14632 (0.58)	
**Alcohol use disorder**			
No	286535 (96.35)	2516677 (98.94)	< 0.001
Yes	10852 (3.65)	26944 (1.06)	
**Schizophrenia**			
No	295256 (99.28)	2535470 (99.68)	< 0.001
Yes	2131 (0.72)	8151 (0.32)	
**Bipolar disorder**			
No	296372 (99.66)	2539463 (99.84)	< 0.001
Yes	1015 (0.34)	4158 (0.16)	
**Total number of psychiatric diagnoses**			
0	276160 (92.86)	2465725 (96.94)	< 0.001
1	13913 (4.68)	58375 (2.29)	
2	5529 (1.86)	15546 (0.61)	
3-6	1785 (0.60)	3975 (0.16)	
**Standardized score of the first principal component**			
Mean (SD)	0.22 (1.56)	-0.025 (0.91)	< 0.001

Note. For numeric variables, standard 2-sample t-test was performed. For categorical variables, chi-squared test was performed.

Table S9. Comparison between sibling pairs included and excluded from the final full-sibling subsample

Number of sibling pairs, n (%)	Sibling pairs excluded (N=268847)	Sibling pairs included in the final full-sibling subsample (N=1485880)	*p* value
**Depression**			
Both siblings without	261909 (97.42)	1459603 (98.23)	< 0.001
One with and one without	6807 (2.53)	25861 (1.74)	
Both siblings with	131 (0.05)	416 (0.03)	
**Anxiety spectrum disorder**			
Both siblings without	261478 (97.26)	1458413 (98.15)	< 0.001
One with and one without	7226 (2.69)	27037 (1.82)	
Both siblings with	143 (0.05)	430 (0.03)	
**Drug use disorder**			
Both siblings without	260667 (96.96)	1470908 (98.99)	< 0.001
One with and one without	7803 (2.90)	14559 (0.98)	
Both siblings with	377 (0.14)	413 (0.03)	
**Alcohol use disorder**			
Both siblings without	256079 (95.25)	1455441 (97.95)	< 0.001
One with and one without	12091 (4.50)	29525 (1.99)	
Both siblings with	677 (0.25)	914 (0.06)	
**Schizophrenia**			
Both siblings without	265802 (98.87)	1476562 (99.37)	< 0.001
One with and one without	2987 (1.11)	9175 (0.62)	
Both siblings with	58 (0.02)	143 (0.01)	
**Bipolar disorder**			
Both siblings without	267258 (99.41)	1480971 (99.67)	< 0.001
One with and one without	1578 (0.59)	4881 (0.33)	
Both siblings with	11 (0.00)	28 (0.00)	
**Difference in the total number of psychiatric diagnoses between siblings**			
Mean (SD)	0.14 (0.47)	0.072 (0.33)	< 0.001
**Difference in the standardized score of the first principal component between siblings**			
Mean (SD)	0.67 (1.90)	0.40 (1.28)	< 0.001

Note. For numeric variables, standard 2-sample t-test was performed. For categorical variables, chi-squared test was performed.

Figure S1. Between-individual and between-sibling associations of psychiatric diagnoses with subsequent all-cause dementia and Alzheimer's disease

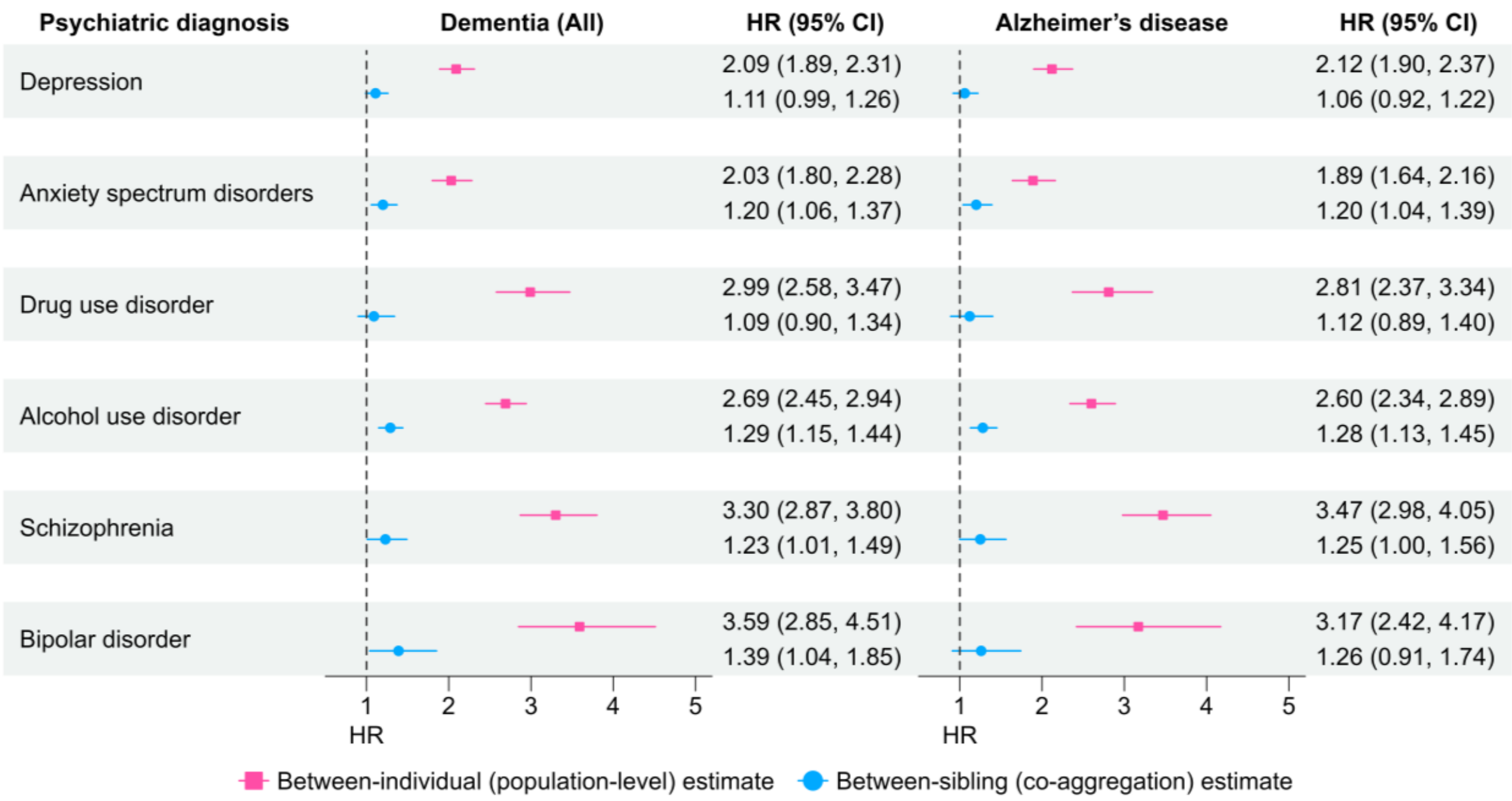

Note. HR: hazard ratio. Between-individual (population-level) estimates were derived from regressions applied to the full sample, adjusted for age, sex, and education level. Between-sibling (co-aggregation) estimates were derived from familial co-aggregation analysis applied to the full-sibling subsample, adjusting for both siblings' age, sex, and education level.

Figure S2. Between-individual and between-sibling associations of latent psychopathology factors with subsequent all-cause dementia and Alzheimer's disease

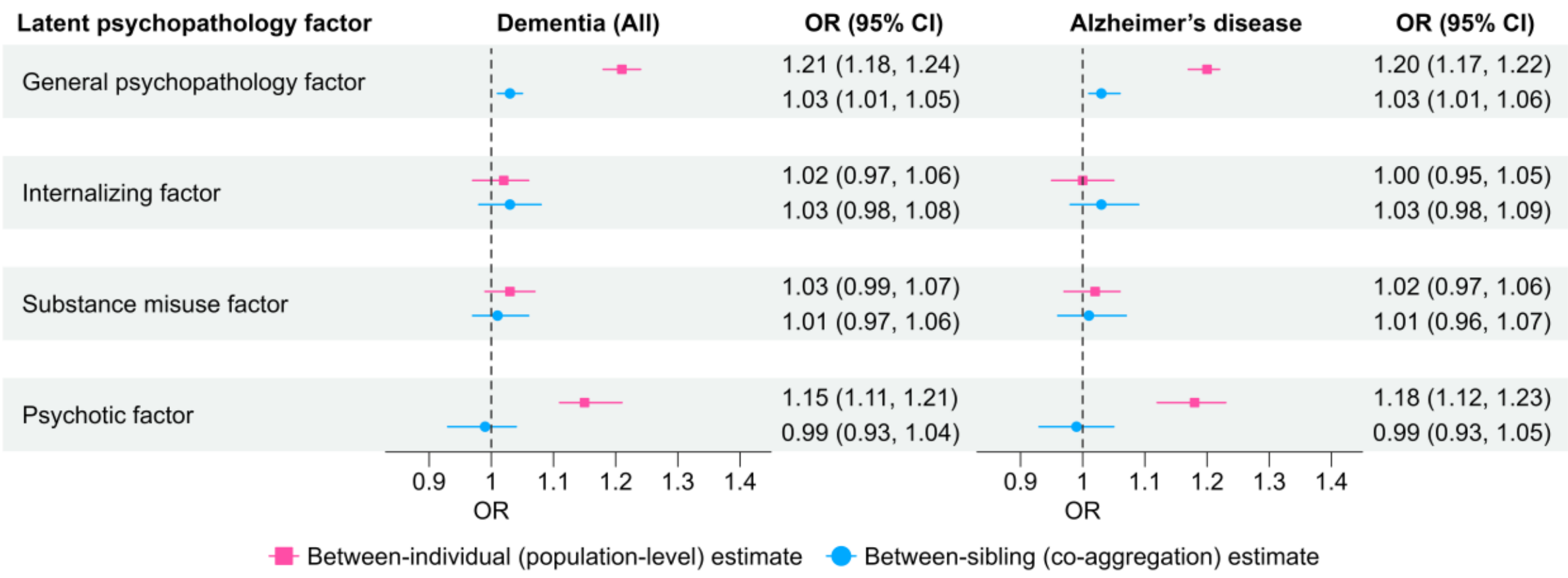

Note. OR: odds ratio. Between-individual (population-level) estimates were derived from regressions applied to the full sample, adjusted for age, sex, and education level. Between-sibling (co-aggregation) estimates were derived from familial co-aggregation analysis applied to the full-sibling subsample, adjusting for both siblings' age, sex, and education level.

Figure S3. Associations of three (one general and two specific) latent psychopathology factors with subsequent all-cause dementia and Alzheimer's disease

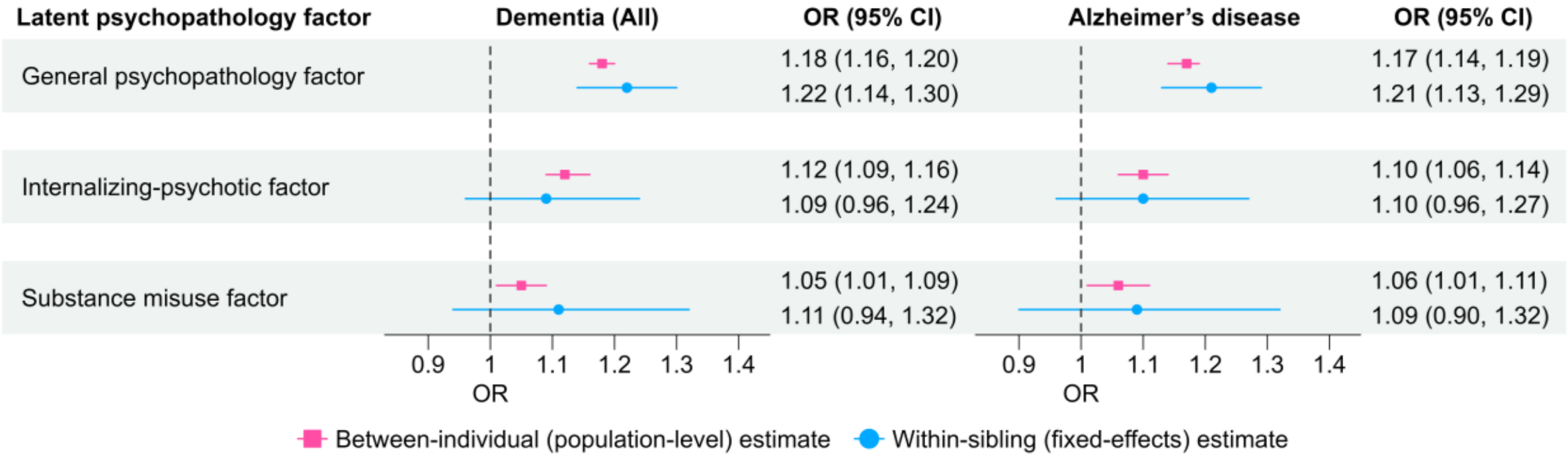

Note. OR: odds ratio. Between-individual (population-level) estimates were derived from regressions applied to the full sample, adjusted for age, sex, and education level. Within-sibling (fixed-effects) estimates were derived from fixed-effects models applied to the full-sibling subsample, adjusting for both measured sibling-varying confounders (age, sex, and education level) and unmeasured sibling-shared confounders (i.e., approximately 50% genetics and 100% shared early-life environment).

Figure S4. Associations of one latent general psychopathology factor with subsequent all-cause dementia and Alzheimer's disease

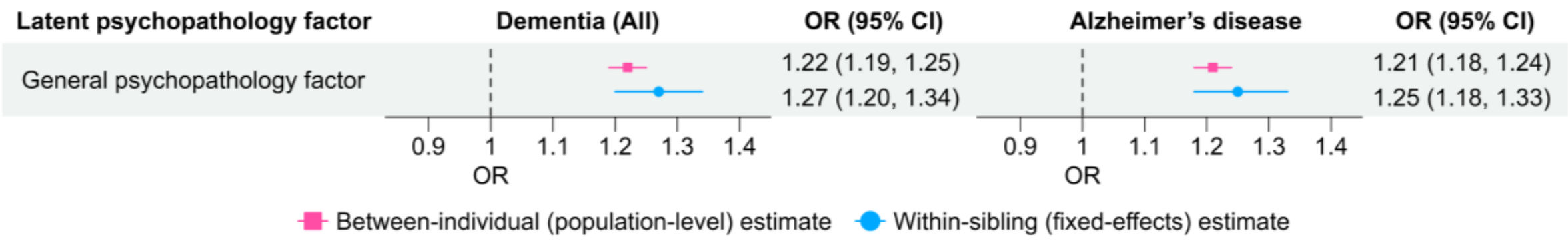

Note. OR: odds ratio. Between-individual (population-level) estimates were derived from regressions applied to the full sample, adjusted for age, sex, and education level. Within-sibling (fixed-effects) estimates were derived from fixed-effects models applied to the full-sibling subsample, adjusting for both measured sibling-varying confounders (age, sex, and education level) and unmeasured sibling-shared confounders (i.e., approximately 50% genetics and 100% shared early-life environment).

Figure S5. Odds ratios for the associations of psychiatric diagnoses with subsequent all-cause dementia and Alzheimer's disease

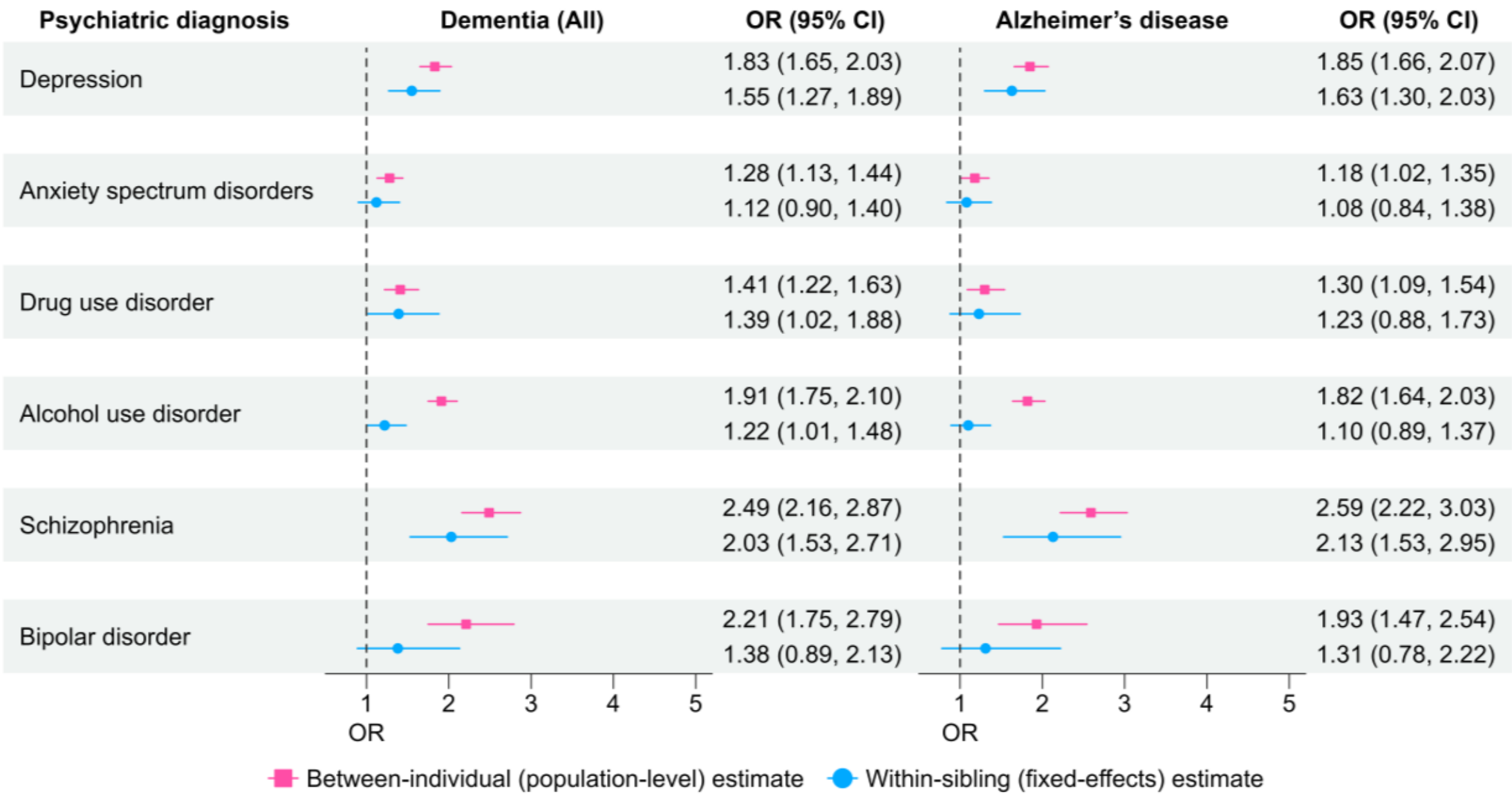

Note. OR: odds ratio. Between-individual (population-level) estimates were derived from regressions applied to the full sample, adjusted for age, sex, and education level. Within-sibling (fixed-effects) estimates were derived from fixed-effects models applied to the full-sibling subsample, adjusting for both measured sibling-varying confounders (age, sex, and education level) and unmeasured sibling-shared confounders (i.e., approximately 50% genetics and 100% shared early-life environment).

Figure S6. Associations of the factor score from the single general-factor model with subsequent all-cause dementia and Alzheimer's disease

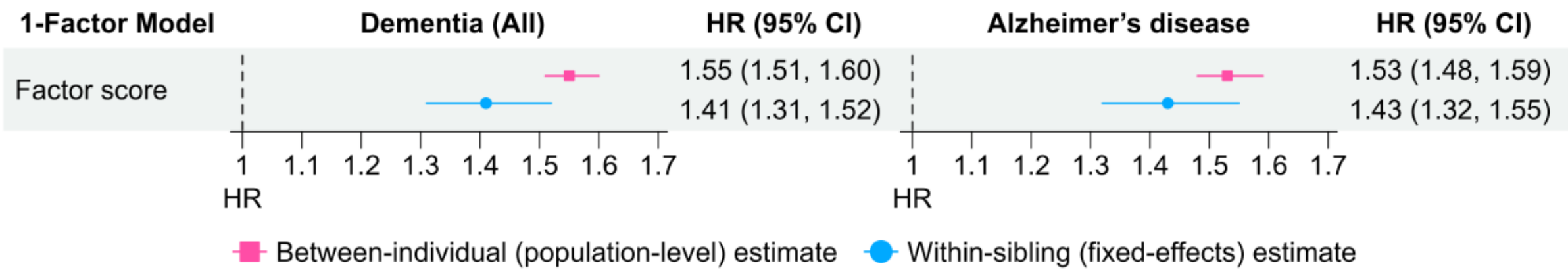

Note. HR: hazard ratio. Between-individual (population-level) estimates were derived from regressions applied to the full sample, adjusted for age, sex, and education level. Within-sibling (fixed-effects) estimates were derived from fixed-effects models applied to the full-sibling subsample, adjusting for both measured sibling-varying confounders (age, sex, and education level) and unmeasured sibling-shared confounders (i.e., approximately 50% genetics and 100% shared early-life environment).

Figure S7. Associations of the standardized score of the first principal component with subsequent all-cause dementia and Alzheimer's disease

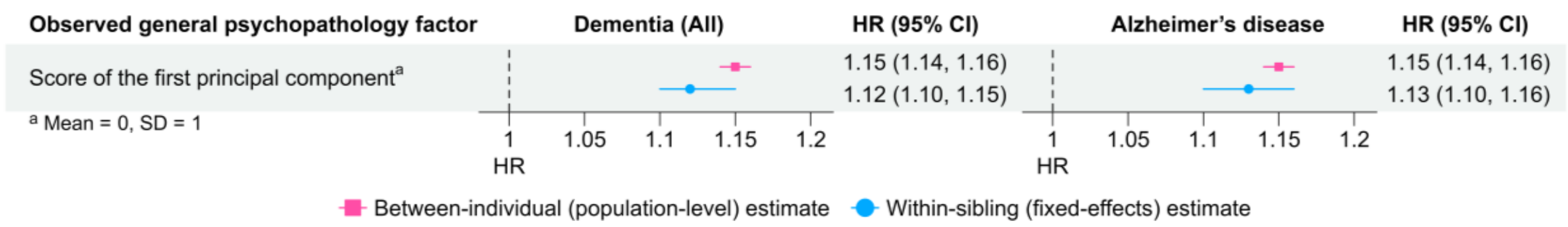

Note. HR: hazard ratio. Between-individual (population-level) estimates were derived from regressions applied to the full sample, adjusted for age, sex, and education level. Within-sibling (fixed-effects) estimates were derived from fixed-effects models applied to the full-sibling subsample, adjusting for both measured sibling-varying confounders (age, sex, and education level) and unmeasured sibling-shared confounders (i.e., approximately 50% genetics and 100% shared early-life environment).

Figure S8. Associations of the total number of psychiatric diagnoses with subsequent all-cause dementia and Alzheimer's disease

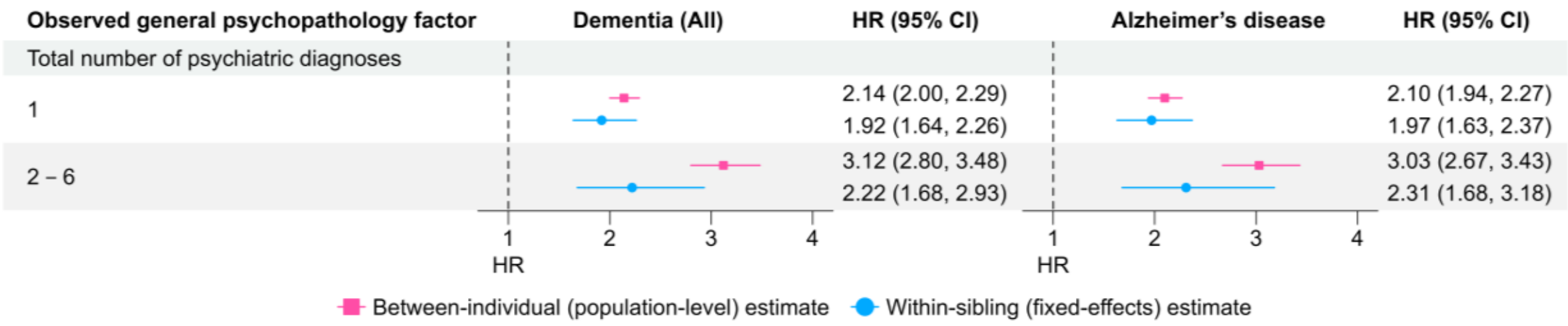

Note. HR: hazard ratio. Between-individual (population-level) estimates were derived from regressions applied to the full sample, adjusted for age, sex, and education level. Within-sibling (fixed-effects) estimates were derived from fixed-effects models applied to the full-sibling subsample, adjusting for both measured sibling-varying confounders (age, sex, and education level) and unmeasured sibling-shared confounders (i.e., approximately 50% genetics and 100% shared early-life environment).

Figure S9. Between-individual associations of psychiatric diagnoses with subsequent all-cause dementia and Alzheimer's disease in the full sample and the full-sibling subsample

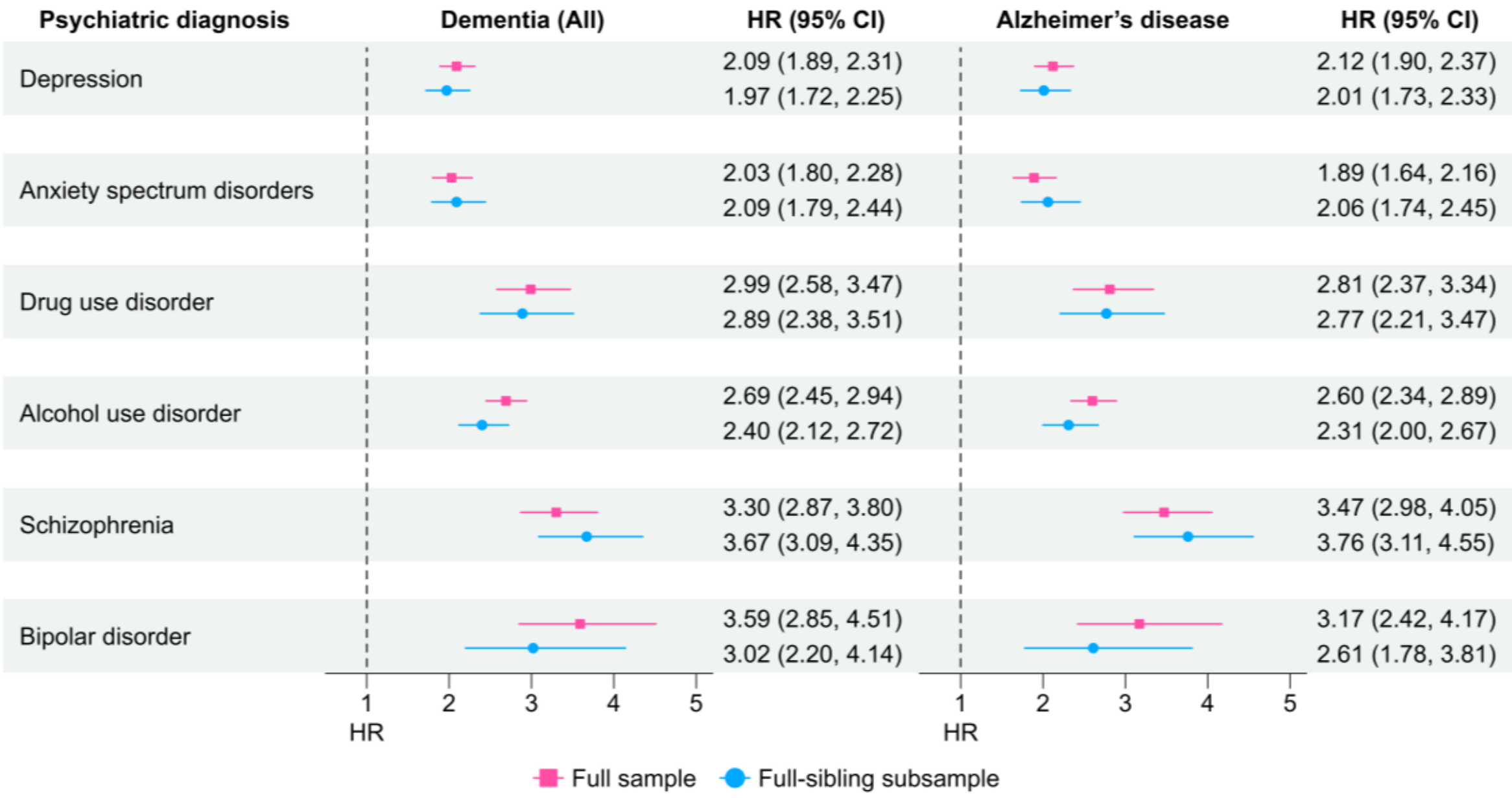

Note. HR: hazard ratio. Between-individual (population-level) estimates were derived from Cox regressions, with attained age as the time scale and adjustment for sex and highest education.

Figure S10. Between-individual associations of psychiatric diagnoses with subsequent all-cause dementia and Alzheimer's disease in males and females

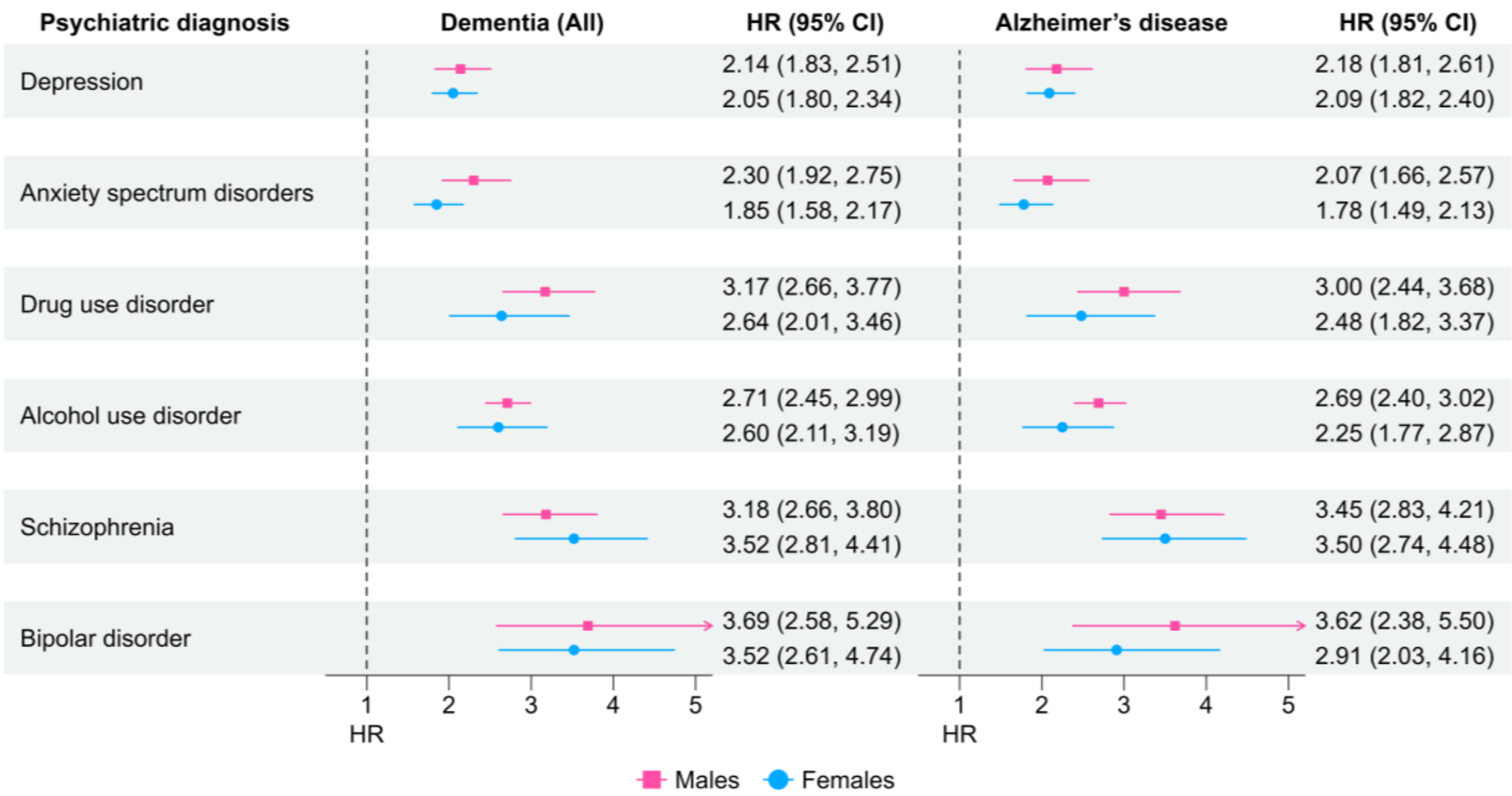

Note. HR: hazard ratio. Between-individual (population-level) estimates were derived from Cox regressions applied to the full sample, with attained age as the time scale and adjustment for sex and education.

Figure S11. Between-individual associations of latent psychopathology factors with subsequent all-cause dementia and Alzheimer's disease in males and females

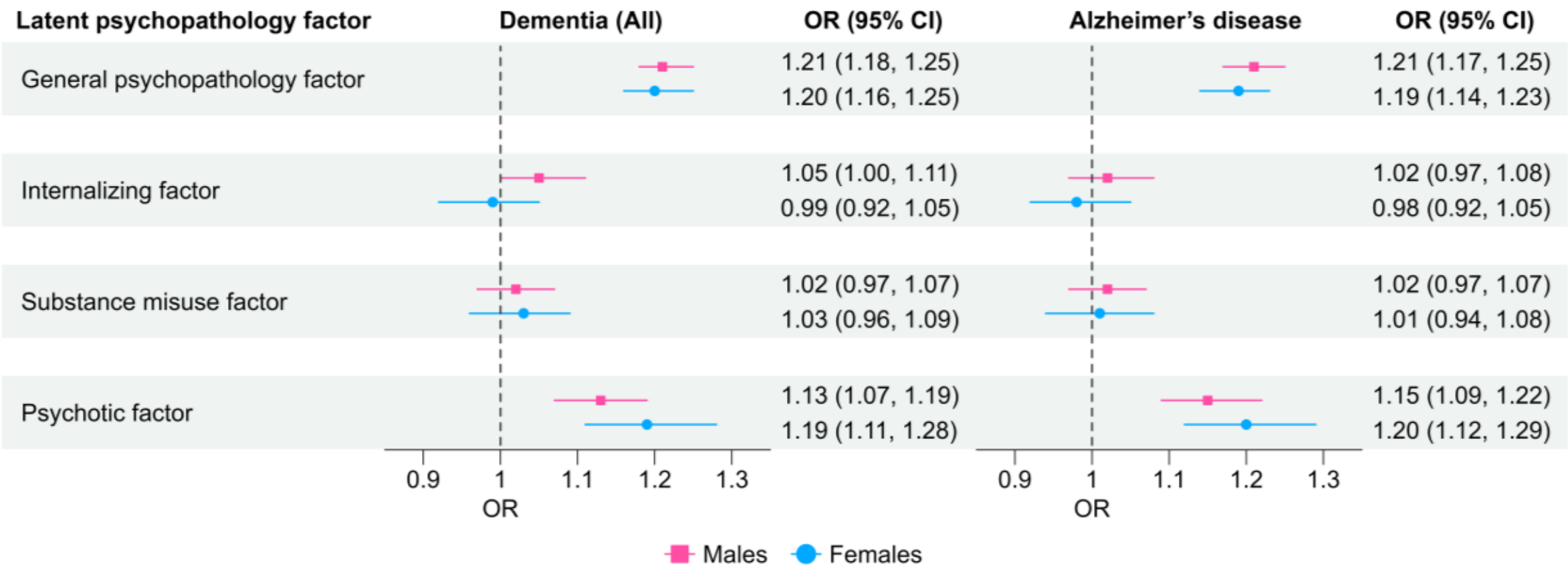

Note. OR: odds ratio. Between-individual (population-level) estimates were derived from regressions applied to the full sample, adjusted for age, sex, and education level.

Figure S12. Associations of psychiatric diagnoses with subsequent early-onset and late-onset all-cause dementia and Alzheimer's disease

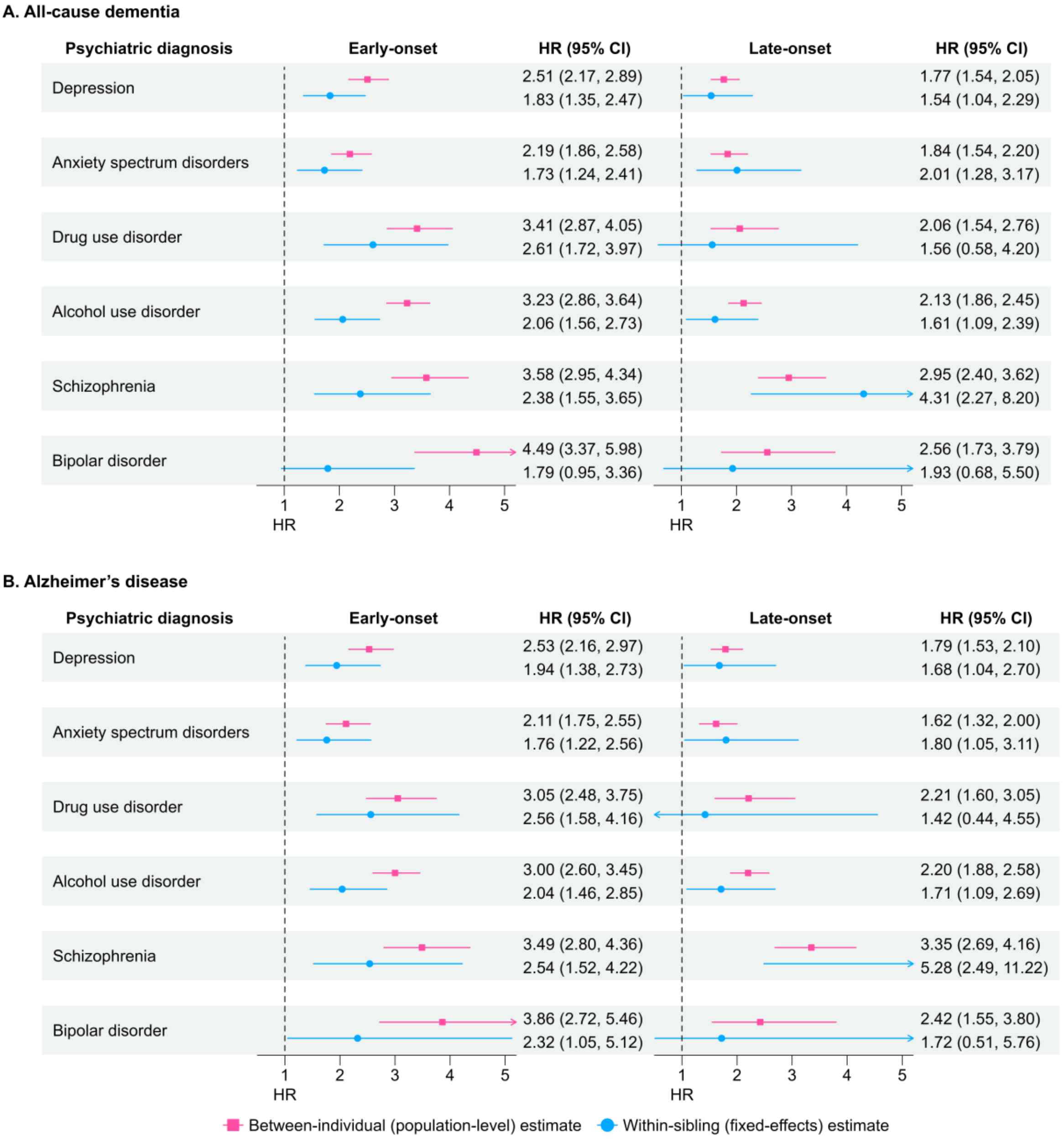

Note. HR: hazard ratio. Between-individual (population-level) estimates were derived from regressions applied to the full sample, adjusted for age, sex, and education level. Within-sibling (fixed-effects) estimates were derived from fixed-effects models applied to the full-sibling subsample, adjusting for both measured sibling-varying confounders (age, sex, and education level) and unmeasured sibling-shared confounders (i.e., approximately 50% genetics and 100% shared early-life environment).

Figure S13. Associations of latent psychopathology factors with subsequent early-onset and late-onset all-cause dementia and Alzheimer's disease

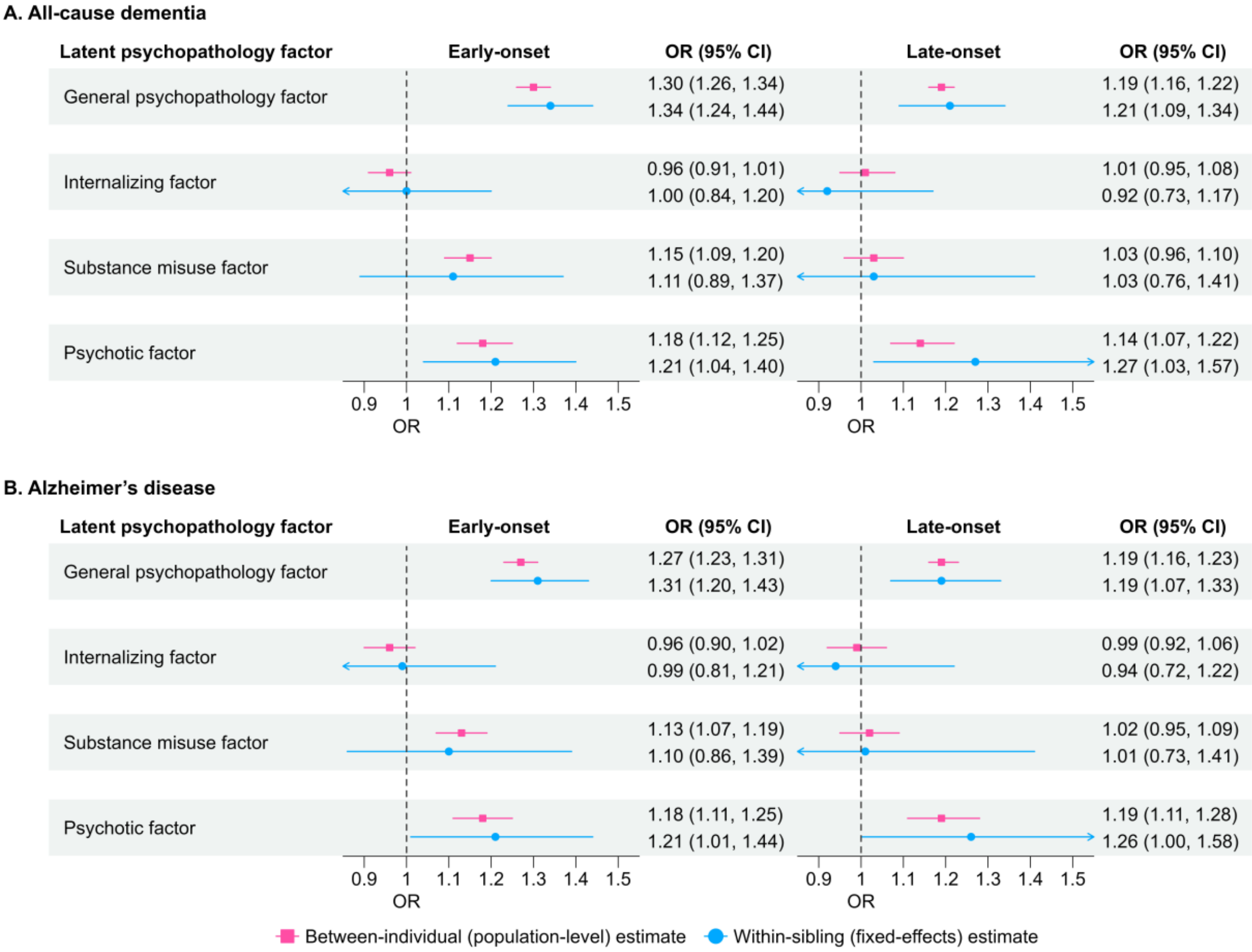

Note. OR: odds ratio. Between-individual (population-level) estimates were derived from regressions applied to the full sample, adjusted for age, sex, and education level. Within-sibling (fixed-effects) estimates were derived from fixed-effects models applied to the full-sibling subsample, adjusting for both measured sibling-varying confounders (age, sex, and education level) and unmeasured sibling-shared confounders (i.e., approximately 50% genetics and 100% shared early-life environment).

Figure S14. Associations of the standardized score of the first principal component by age 35 or 25 years with subsequent all-cause dementia and Alzheimer's disease after age 35, 40, 45, or 50 years

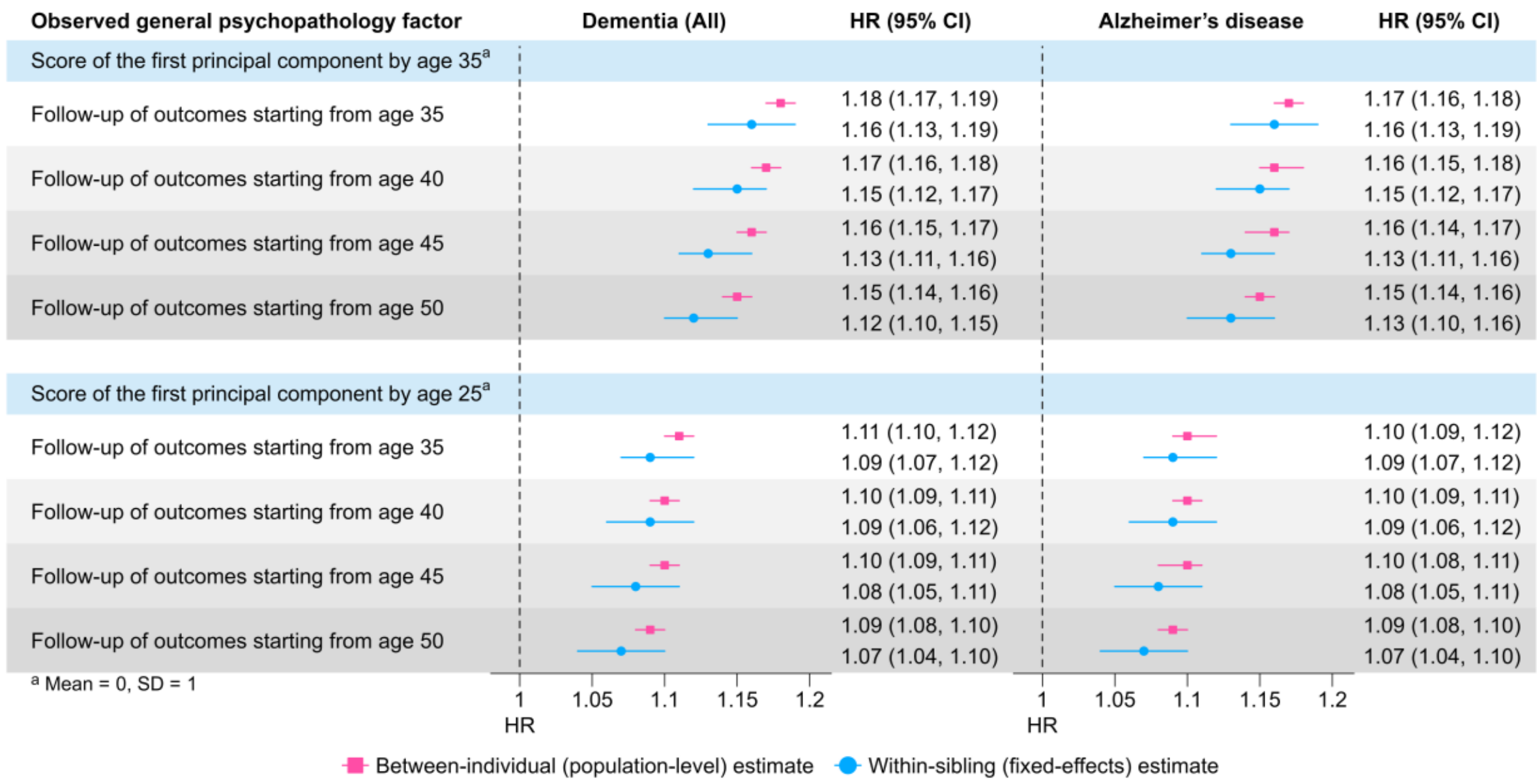

Note. HR: hazard ratio. Between-individual (population-level) estimates were derived from regressions applied to the full sample, adjusted for age, sex, and education level. Within-sibling (fixed-effects) estimates were derived from fixed-effects models applied to the full-sibling subsample, adjusting for both measured sibling-varying confounders (age, sex, and education level) and unmeasured sibling-shared confounders (i.e., approximately 50% genetics and 100% shared early-life environment).

Figure S15. Associations between psychiatric diagnoses and subsequent all-cause dementia and Alzheimer's disease (without adjustment for educational level)

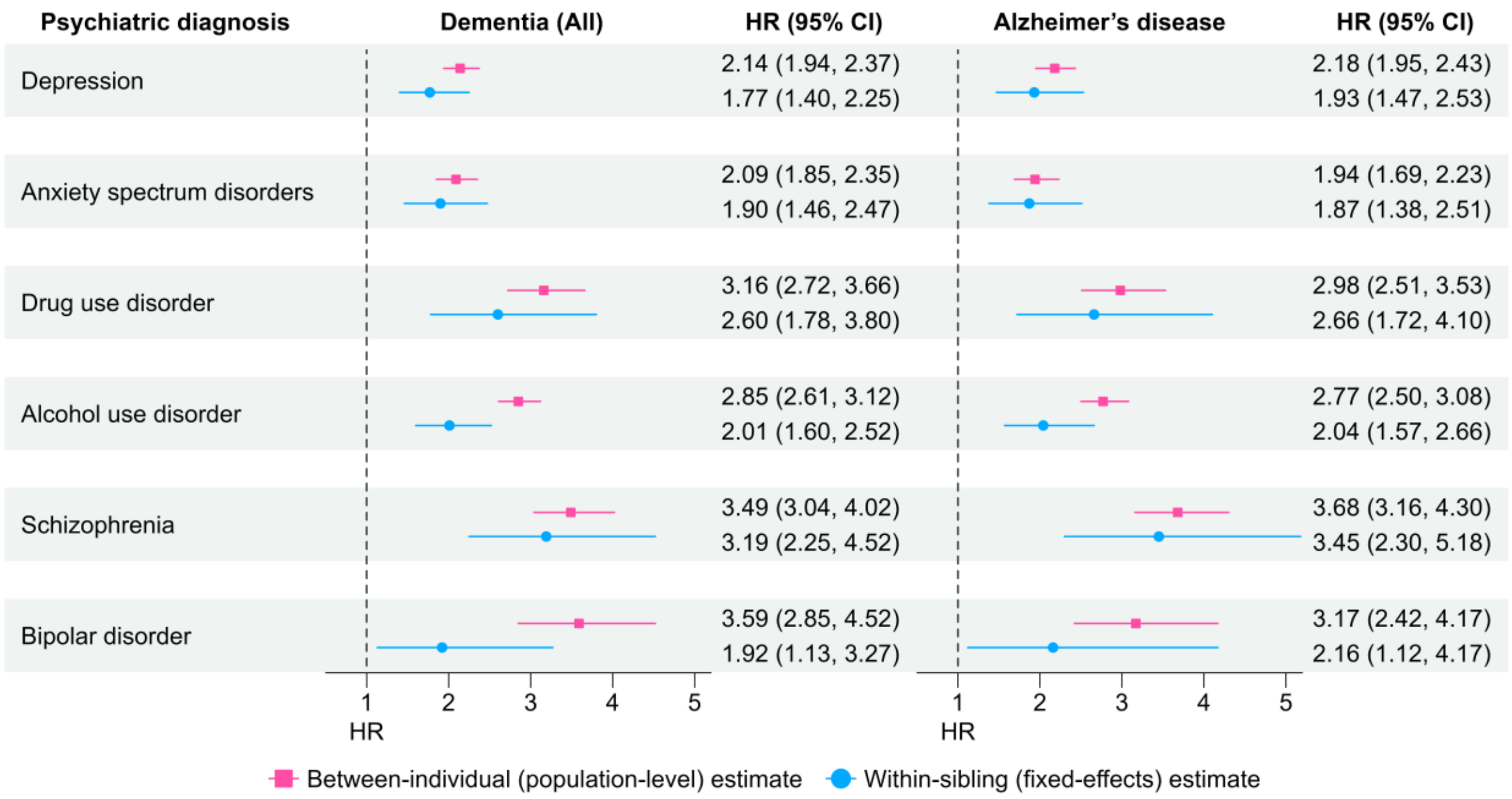

Note. HR: hazard ratio. Between-individual (population-level) estimates were derived from regressions applied to the full sample, adjusted for age and sex. Within-sibling (fixed-effects) estimates were derived from fixed-effects models applied to the full-sibling subsample, adjusting for both measured sibling-varying confounders (age and sex) and unmeasured sibling-shared confounders (i.e., approximately 50% genetics and 100% shared early-life environment).

Figure S16. Associations between latent psychopathology factors and subsequent all-cause dementia and Alzheimer's disease (without adjustment for educational level)

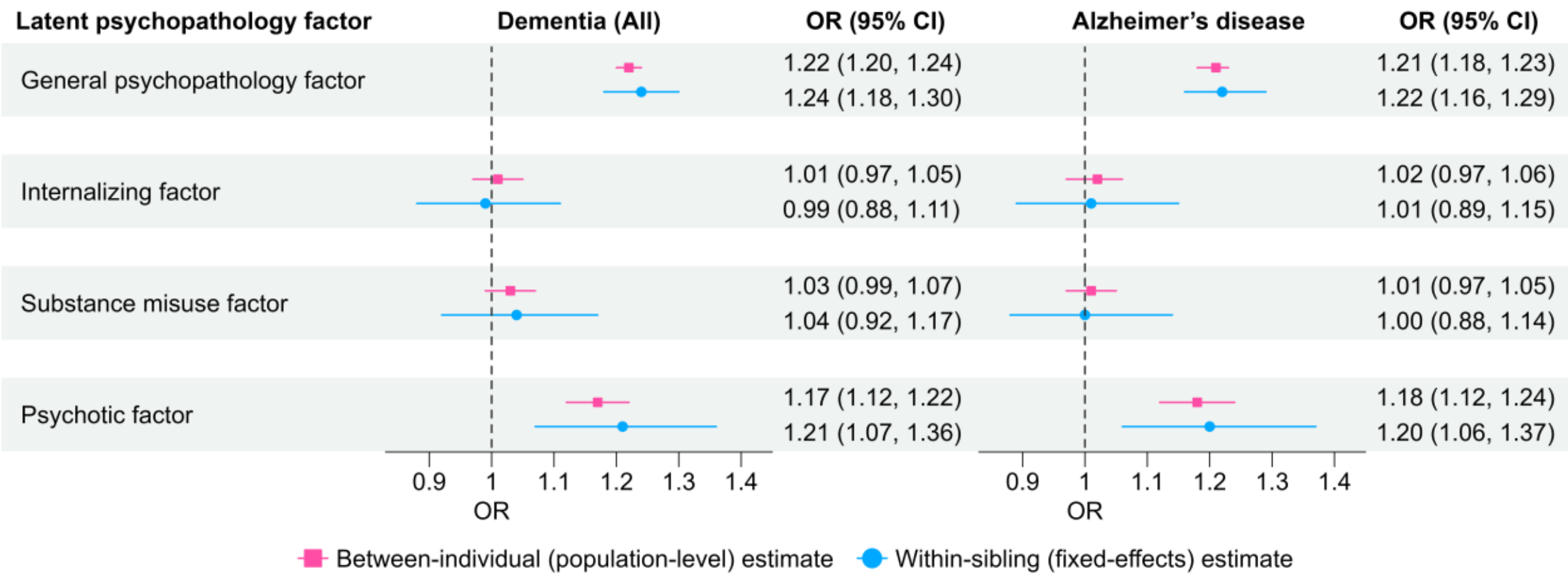

Note. OR: odds ratio. Between-individual (population-level) estimates were derived from regressions applied to the full sample, adjusted for age and sex. Within-sibling (fixed-effects) estimates were derived from fixed-effects models applied to the full-sibling subsample, adjusting for both measured sibling-varying confounders (age and sex) and unmeasured sibling-shared confounders (i.e., approximately 50% genetics and 100% shared early-life environment).

Figure S17. Associations between additional psychiatric disorder groups and subsequent all-cause dementia and Alzheimer's disease

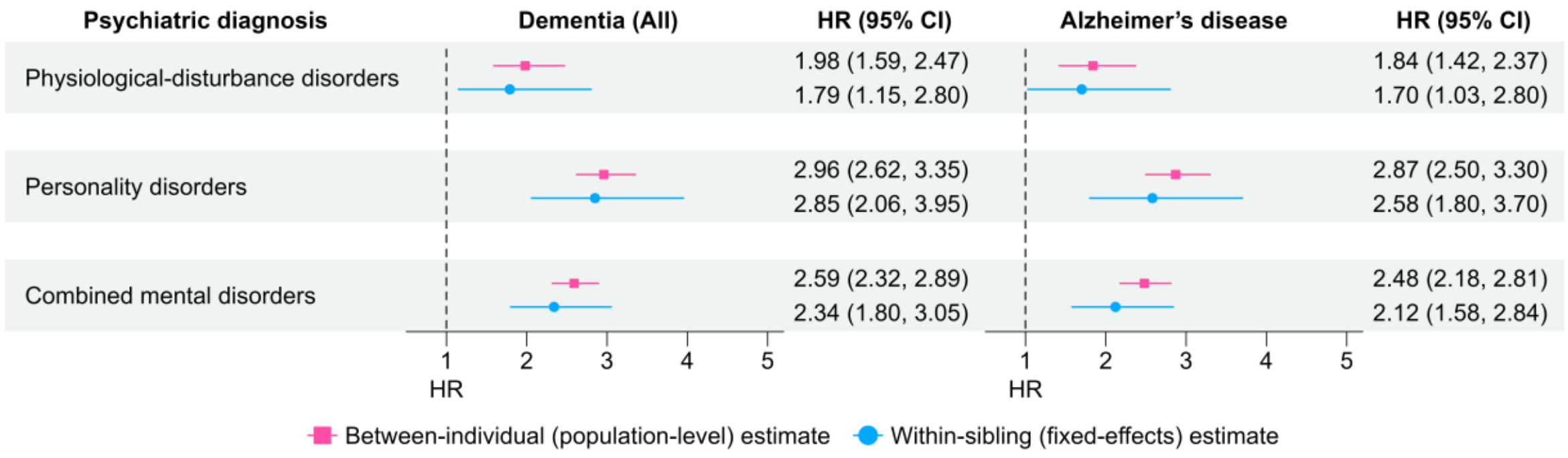

Note. The group "combined mental disorders" contains physiological-disturbance disorders, personality disorders, developmental disorders, behavioral disorders, and unspecified mental disorders. HR: hazard ratio. Between-individual (population-level) estimates were derived from regressions applied to the full sample, adjusted for age, sex, and education level. Within-sibling (fixed-effects) estimates were derived from fixed-effects models applied to the full-sibling subsample, adjusting for both measured sibling-varying confounders (age, sex, and education level) and unmeasured sibling-shared confounders (i.e., approximately 50% genetics and 100% shared early-life environment).
